# Mechanistic Insights into Active Components of Rosa Roxburghii Juice Against Fluoride-Induced Osteoarthritis

**DOI:** 10.3390/antiox15030309

**Published:** 2026-02-28

**Authors:** Youqi Du, Youwen Du, Shaobo Liu, Jun Li, Lianqing Tian, Longyu Yao, Jiajia Liao, Lingyun Fu, Yan Chen, Peng Luo, Xiangchun Shen

**Affiliations:** 1 The State Key Laboratory of Functions and Applications of Medicinal Plants, School of Pharmaceutical Sciences Guizhou Medical University, No. 6 Ankang Avenue, Guiyang 561113, China; 2 The Department of Pharmacology of Materia Medica (The High Efficacy Application of Natural Medicinal Resources Engineering Center of Guizhou Province and The High Educational Key Laboratory of Guizhou Province for Natural Medicinal Pharmacology and Druggability), School of Pharmaceutical Sciences, Guizhou Medical University, No. 6 Ankang Avenue, Guiyang 561113, China; 3 The Key Laboratory of Optimal Utilization of Natural Medicine Resources (The Union Key Laboratory of Guiyang City-Guizhou Medical University), School of Pharmaceutical Sciences, Guizhou Medical University, No. 6 Ankang Avenue, Guiyang 561113, China; 4 Guizhou International Joint Research Center for Druggability Evaluation of Natural Medicines, Guizhou Medical University, No. 6 Ankang Avenue, Guiyang 561113, China; 5 Key Laboratory of Environmental Pollution and Disease Monitoring (Ministry of Education), School of Public Health and Health Sciences, Guizhou Medical University, No. 6 Ankang Avenue, Guiyang 561113, China

**Keywords:** fluoride toxicity, osteoarthritis, oxidative stress, p53 signaling, proteasomal degradation, rosa roxburghii juice, polyphenols, medicine–food homology, network pharmacology, molecular dynamics, ligand fishing mass spectrometry (LiP-MS), inflammation, cytoprotection

## Abstract

Fluoride-induced osteoarthritis (F-OA) is a debilitating manifestation of endemic fluorosis, with limited preventive or therapeutic strategies. Rosa roxburghii juice (RRJ), a traditional medicinal/edible product, has shown protective effects against skeletal fluorosis, yet its active constituents and molecular mechanisms are not fully understood. In this study, an integrated strategy combining bioinformatics analysis, network pharmacology, molecular docking and dynamics simulations, limited proteolysis–mass spectrometry (LiP–MS), and in vitro experiments was employed to systematically elucidate the protective mechanisms of RRJ against F-OA. Forty-four core F-OA-associated genes were identified, with TP53 and the p53 signaling pathway emerging as central regulatory hubs. Quercetin, Epicatechin, Emodin, and Ellagic acid were screened as key bioactive components of RRJ and demonstrated strong binding affinity toward core targets, including TP53. Cellular experiments showed that these compounds significantly attenuated sodium fluoride-induced cellular injury. LiP–MS analysis further revealed widespread protein conformational remodeling following treatment, with TP53 exhibiting pronounced structural sensitivity. Mechanistically, these active compounds mitigated fluoride-induced pathological changes by suppressing p53 mRNA expression and restoring proteasome-mediated p53 degradation. This study provides systematic pharmacological evidence supporting Rosa roxburghii fruit as a promising functional food for the prevention and management of skeletal fluorosis and F-OA.

## 1. Introduction

Chronic systemic fluorosis is induced by prolonged and excessive fluoride intake, with skeletal fluorosis representing the most severe clinical subtype. Excessive fluoride accumulates in mineralized tissues and progressively disrupts bone homeostasis, resulting in osteosclerosis, joint pain, restricted mobility, skeletal deformity, and, in advanced stages, neurological compression-related paralysis [1]. Dental fluorosis is widely recognized as the earliest biomarker of fluoride overexposure; however, skeletal fluorosis reflects much deeper metabolic damage characterized by dysregulated osteoblast and osteoclast activity, oxidative stress, inflammatory injury, and impaired bone remodeling [2,3]. When fluoride toxicity involves articular tissues, secondary osteoarthritis frequently develops as a major clinical manifestation. Unlike primary osteoarthritis, which is usually driven by age-related degeneration, mechanical overload, or obesity, fluoride-induced osteoarthritis (F-OA) originates from chronic toxic insult. Recent mechanistic studies have demonstrated that fluoride disrupts cartilage extracellular matrix homeostasis, induces chondrocyte apoptosis, alters subchondral bone turnover, and upregulates catabolic mediators such as matrix metalloproteinase-13 (MMP-13) and inflammatory cytokine [4,5]. Due to its toxic etiology, conventional symptomatic treatments such as NSAIDs or intra-articular corticosteroids provide limited therapeutic benefit, highlighting the need for multi-target intervention strategies capable of attenuating fluoride-related oxidative, inflammatory, and metabolic disturbances.

Bioinformatics and network pharmacology demonstrate powerful complementary strengths for uncovering the deep toxicological mechanisms by which fluoride induces osteoarthritis. They can systematically integrate massive multi-omics data—from the genome and transcriptome to the metabolome—and, through efficient data mining and network analysis, reconstruct the full chain of “fluoride exposure–molecular targets–signaling pathways–pathological phenotypes.” This system-level mapping guides multi-target intervention strategies and thus enables a holistic dissection of the complex network mechanisms underlying fluorosis-associated osteoarthritic lesions [6]. Rosa roxburghii Tratt (RRT), a fruit belonging to the Rosaceae family, is indigenous to southwestern China, serves as a significant local agricultural product, and is traditionally used to aid digestion. Its fruit is rich in flavonoids, polyphenols, triterpenes, and vitamin C and exhibits marked antioxidant, anti-inflammatory, and cytoprotective activities. With both notable medicinal properties and edibility, it serves as a representative example of a medicine–food homology (MFH) substance [7]. Moreover, recent studies report that Rosa roxburghii juice (RRJ) has strong antioxidant effects and can improve indicators of liver and kidney function, protect collagenous tissues, and modulate the gut-microbiota–gut–bone axis; these findings together point to the therapeutic and development potential of RRJ against endemic fluorosis-related skeletal disease, including fluoride-induced osteoarthritis [8,9]. Accordingly, an integrated pharmacological approach was adopted to elucidate RRJ’s protective mechanisms against F-OA, alongside a preliminary in-depth exploration of its bioactive components and underlying actions. Through bioinformatic prediction, network toxicology/pharmacology analyses, molecular docking, liquid chromatography–mass spectrometry (Lip-MS), ligand fishing, and molecular dynamics simulations, we constructed an interaction network between the active constituents of RRJ and OA-related targets, followed by validation using Rat synovial fibroblast models to confirm the computational findings. This study is the first to systematically demonstrate the potential of RRJ as a multi-component, multi-target MFH intervention for F-OA, thereby laying the groundwork for subsequent translational studies and fostering the advancement of therapeutic or functional food products for skeletal fluorosis.

## 2. Materials and Methods

### 2.1. Materials and Reagents

Sodium fluoride standard solution (S885178) and ELISA kits for TNF-α, IL-1β, IL-6, and IL-10 were sourced from Macklin Biochemical Technology Co., Ltd. (Shanghai, China) and Shanghai Fanyin Biotechnology Co., Ltd. (Shanghai, China), respectively. The Reactive Oxygen Species (ROS) Assay Kit (S0033S) was obtained from Beyotime Biotechnology Co., Ltd. (Shanghai, China). The Cell Cycle Detection Kit (KGA9101) was purchased from KeyGEN BioTECH Co., Ltd. (Nanjing, China). The antibodies used in the experiment included the following: anti-P53 (10442-1-AP), anti-P21 (28248-1-AP), and anti-P-P53 (67826-1-lg) were all procured from Wuhan Sanying Biotechnology Co., Ltd. (Wuhan, China); anti-α-tubulin (ER130905) was acquired from Hangzhou Hua’an Biotechnology Co., Ltd. (Hangzhou, China); and Rat synovial fibroblast cells (iCell-0135a) and the cell culture medium (11095080) were obtained from iCell Bioscience Inc. (Shanghai, China) and Thermo Fisher Scientific Inc. (Waltham, MA, USA), respectively. The RNA-easy Isolation Reagent kit was purchased from Vazyme Biotech Co., Ltd. (Nanjing, China). The cDNA Synthesis SuperMix kit and PerfectStart Green qPCR SuperMix were procured from TransGen Biotech Co., Ltd. (Beijing, China). Emodin (C_15_H_10_O_5_, CAS 518-82-1, HPLC ≥ 98%), Quercetin (C_15_H_10_O_7_, CAS 117-39-5, HPLC ≥ 98%), Epicatechin (C_15_H_14_O_6_, CAS 490-46-0, HPLC ≥ 98%), and Ellagic acid (C_14_H_6_O_8_, CAS 476-66-4, HPLC ≥ 98%) were purchased from Shanghai Yuanye Bio-Technology Co., Ltd. (Shanghai, China).

### 2.2. Bioinformatic Data Download and Preprocessing

In this study, microarray datasets were analyzed to obtain gene expression profiles and clinical phenotypic information associated with the keywords “osteoarthritis,” “Homo sapiens,” and “gene expression.” All relevant gene expression datasets and platform annotation files were accessed from the publicly available Gene Expression Omnibus (GEO) repository (https://www.ncbi.nlm.nih.gov/geo/). As such, this study did not require separate ethical approval or informed consent. To ensure analytical consistency, two datasets (GSE82107 and GSE206848) sharing the GPL570-55999 platform were selected. GSE82107 comprised samples from 7 healthy controls and 10 OA patients, and GSE206848 included 7 controls alongside 9 OA patients.

To obtain accurate mRNA expression profiles, Perl scripts were used to match and align the transcriptomic data from GSE82107 and GSE206848. The sva package (version 3.50.0) in R (version 4.3.2) [10] was employed to identify and correct batch effects using its default parameter settings. Background correction was applied using the robust multi-array average (RMA) method [11] to ensure data consistency across platforms. The ComBat algorithm was subsequently employed to further eliminate batch effects between samples. The processed data were transformed using a natural logarithm to optimize data distribution and enhance analytical performance. Post-correction expression data were visualized using box plots and principal component analysis (PCA), yielding a final dataset of 14 control samples and 17 OA samples for downstream analysis.

Furthermore, to explore pathogenic genes associated with OA, we retrieved information on genes known to be involved in OA progression (as of October 2024) from the Comparative Toxicogenomics Database (CTD, http://ctdbase.org). Candidate genes related to sodium fluoride were predicted by integrating data from ChEMBL (https://www.ebi.ac.uk/chembl/), STITCH (http://stitch.embl.de/), PubChem (https://pubchem.ncbi.nlm.nih.gov/), and the CTD database.

### 2.3. Identification and Processing of Co-Expressed Genes Related to OA and Fluorosis

To identify differentially expressed genes (DEGs) between osteoarthritis (OA) samples and healthy controls, differential expression analysis was conducted on the integrated dataset using the R package limma (version 3.58.1) [12]. We defined statistical significance using a *p*-value cutoff of 0.05 and required an absolute log_2_ fold change (|log_2_FC|) of more than 0.585 for gene selection. Subsequently, high-confidence OA-associated genes with an Inference Score > 10 were obtained from the CTD. These genes were merged with sodium fluoride-associated genes predicted from STITCH, ChEMBL, PubChem, and CTD databases. After removing duplicates, a candidate gene set related to fluoride toxicity was compiled. The intersection among OA-related DEGs, key OA genes, and fluoride-related genes was then defined, yielding a set of candidate genes potentially linking OA to fluorosis.

The expression patterns of DEGs were visualized through heatmaps using the ggplot2 (version 4.0.1) and pheatmap (version 1.0.13) packages [13]. To further explore gene-gene correlations, the corrplot package was used to calculate Pearson correlation coefficients, helping to uncover potential regulatory modules and network structures. These overlapping target genes were then input into the STRING database [14] (https://cn.string-db.org/) for protein-protein interaction (PPI) analysis. The resulting network was imported into Cytoscape software (version 3.8.2) [15] for further topological analysis and hub gene identification.

### 2.4. Pathway and Functional Profiling of Fluorosis-Related OA Genes

To elucidate the biological roles and pathways of the candidate genes, we conducted Gene Ontology (GO) and Kyoto Encyclopedia of Genes and Genomes (KEGG) enrichment analyses with the R package clusterProfiler (version 4.10.1) [16]. A *p* value of < 0.05 was considered statistically significant. This analysis sought to clarify the biological processes and signaling pathways by which sodium fluoride may contribute to the initiation and progression of osteoarthritis (OA), offering preliminary mechanistic insights.

### 2.5. Preparation of Rosa Roxburghii Juice (RRJ)

Fresh fruits of Rosa roxburghii Tratt. cultivar ‘Guinong 5’ were purchased from the Jinsha area of Bijie City, Guizhou Province, China. The plant material was authenticated as fresh fruits of the current season by the School of Pharmacy, Guizhou Medical University. A photograph of the representative fruit samples used in this study is provided in Supplementary Figure S1. After collection, the fruits were placed in sealed plastic ziplock bags, transported to the laboratory in a portable refrigerated container maintained at 4 °C, thoroughly washed with distilled water, and air-dried. The juice was extracted using a juice extractor, and the freshly obtained juice was directly used for subsequent analyses and is hereafter referred to as Rosa roxburghii juice (RRJ).

Sample preparation for LC–MS analysis: A 600 μL aliquot of the RRJ suspension was transferred into a 1.5 mL EP tube, followed by the addition of 400 μL of methanol. The mixture was vortexed for 10 s, after which 200 μL of the resulting solution was mixed with 200 μL of 40% (*v*/*v*) aqueous methanol and vortexed again for 10 s. For LC-MS analysis, samples were centrifuged at 16,000× *g* for 15 min at 4 °C, and the supernatant was collected for analysis. For HPLC analysis, the sample was prepared by diluting 1 mL of the RRJ suspension to 10 mL with 70% (*v*/*v*) methanol in a volumetric flask. After standing, the supernatant was similarly collected for HPLC analysis.

### 2.6. Establishment of LC–MS and HPLC Analytical Methods for RRJ

LC–MS analysis: Chemical profiling of RRJ was performed using an ultra-high-performance liquid chromatography (UHPLC) system coupled to a Q-Exactive HFX mass spectrometer (Thermo Fisher Scientific, Waltham, MA, USA). Chromatographic separation was achieved at a column temperature of 35 °C with a flow rate of 0.3 mL/min. The mobile phase consisted of 0.1% formic acid in water (A) and 0.1% formic acid in acetonitrile (B). Gradient elution was conducted according to the program shown in Table 1. Mass spectrometric detection was carried out using an electrospray ionization (ESI) source operated in both positive and negative ion modes. Data acquisition was performed in Full-MS/dd-MS^2^ mode with resolutions set to 60,000 (MS^1^) and 15,000 (MS^2^). The stepped normalized collision energies were 20, 40, and 60, and the MS^1^ scan range covered *m*/*z* 90 to 1300.

For quantitative analysis, an HPLC-DAD method was developed using a Dionex Ultimate 3000 system (Thermo Fisher Scientific, Waltham, MA, USA). Chromatographic separation was achieved on a Gemini NX-C18 reversed-phase column (Phenomenex) maintained at 30 °C, with an injection volume of 5 μL. The mobile phase consisted of solvent A (0.1% phosphoric acid in water) and solvent B (acetonitrile), applied under a linear gradient program over 30 min.

For ellagic acid analysis, the gradient was programmed from 80% A/20% B to 70% A/30% B, with detection at 254 nm. For epicatechin determination, the gradient was adjusted from 85% A/15% B to 80% A/20% B, and detection was performed at 280 nm. Reference standards of ellagic acid (23 mg/mL) and epicatechin (68.6 mg/mL) were dissolved in methanol to prepare stock solutions.

Method validation was conducted in accordance with the International Council for Harmonisation guideline ICH Q2(R1). Linearity was evaluated at six concentration levels for each analyte by constructing calibration curves of peak area versus concentration. The corresponding calibration curves are shown in Supplementary Figure S2A (epicatechin) and Supplementary Figure S2B (ellagic acid). The limits of detection (LODs) and quantification (LOQs) were determined based on signal-to-noise ratios of 3 and 10, respectively, and are illustrated in Supplementary Figure S2C–F.

Precision was assessed through intra-day and inter-day analyses at three concentration levels, while repeatability was evaluated using six independently prepared sample solutions (Supplementary Table S1). Accuracy was determined by recovery experiments using the standard addition method at spiking levels of 80%, 100%, and 120%, with results presented in Supplementary Table S2. Comprehensive validation parameters, including regression equations, correlation coefficients (R^2^), LODs, LOQs, precision (RSD%), and recovery values, are summarized in Supplementary Table S3.

### 2.7. Network Pharmacology Analysis

The active compounds present in RRJ were sourced based on a comprehensive review of previously published literature [17] and their molecular structure information was retrieved from the PubChem database. Potential targets of these compounds were predicted using the SwissTargetPrediction platform [18,19] (https://www.swisstargetprediction.ch/) and the Traditional Chinese Medicine Systems Pharmacology Database and Analysis Platform (TCMSP) (https://www.tcmsp-e.com/load_intro.php?id=43). These predicted targets were then intersected with the previously identified fluorosis-related osteoarthritis (OA) gene set to obtain shared potential regulatory targets.

The shared targets were submitted to the STRING database to construct a protein–protein interaction (PPI) network. A Sankey diagram of “drug-compound-target-disease” relationships was constructed using the Microbioinformatics platform. Additionally, GO functional annotation and KEGG pathway enrichment analyses of the common targets were conducted using the clusterProfiler package in R [16] to investigate the biological processes and signaling pathways potentially underlying the effects of RRJ on fluorosis-associated osteoarthritis (OA). Enrichment outcomes were visualized using bubble plots, with statistical significance defined as *p* < 0.05.

### 2.8. Molecular Docking

Compound structures were retrieved from PubChem (https://pubchem.ncbi.nlm.nih.gov/) and converted to PDBQT format using Open Babel 2.3.2. Crystal structures of the target proteins, including AKT1 (7MYX), BAX (8SRX), FGR (7JT9), FOS (1A02), HP (4 × 0 L), IL1A (5UC6), MMP12 (1RMZ), PTGS1 (6Y3C), and TP53 (3DCY), were obtained from the Protein Data Bank (PDB).

All receptor proteins underwent structural preparation in PyMOL 2.3.0, including removal of solvent molecules and existing ligands, after which hydrogen addition and charge assignment were performed with AutoDockTools. The finalized protein models were exported in PDBQT format for subsequent docking analysis. Receptor–ligand molecular docking was executed using AutoDock Vina 1.5.7 [20]. Binding interactions were characterized using the Protein–Ligand Interaction Profiler (PLIP) [21] (https://plip-tool.biotec.tu-dresden.de/plip-web/plip/index), while docking poses were rendered with PyMOL. Two-dimensional representations of protein–ligand interactions were generated using Discovery Studio 2019.

### 2.9. Molecular Dynamics (MD) Simulation and Binding Free Energy Calculation

Molecular dynamics (MD) simulations were conducted using GROMACS 2022.3 [22]. The CHARMM36 force field (charmm36-jul2022.ff) was applied to generate topology files for the TP53 protein. The simulation system was solvated with the TIP3P water model, and Na^+^ and Cl^−^ ions were introduced to ensure overall charge neutrality. Equilibration was performed under both NVT (constant volume and temperature) and NPT (constant pressure and temperature) ensembles for 100 ps at 300 K. This was followed by an 80 ns production MD simulation for each protein–ligand complex using a 2 fs integration time step.

To investigate the binding affinity between ligands (Quercetin, Ellagic Acid, Epicatechin, and Emodin) and TP53, the molecular mechanics/Poisson-Boltzmann surface area (MM/PBSA) method was applied. The binding free energy was calculated using the gmx_MMPBSA (version 1.9.1) [23] module (Python-based interface) from the GROMACS suite [24]. The total binding free energy was derived from the difference between the free energy of solvation and the molecular mechanical potential energy of the complex.

Furthermore, the binding energy contributions of individual amino acid residues at the protein-ligand interface were analyzed to identify key residues involved in stabilizing the complexes with TP53.

### 2.10. Sample Preparation and Extraction in Limited Proteolysis Mass Spectrometry (Lip-MS) Analysis

Rat knee synovial fibroblasts were homogenized and proteins were extracted using cold 1× PBS, with protein quantification performed by BCA assay. After activation, cells were randomly divided into untreated and drug-treated groups (treated with a mixture of 5 μM Quercetin, 20 μM Epicatechin, 5 μM Emodin, and 1 μM Ellagic acid). During drug treatment, the control group received the corresponding solvent.

The treated samples were digested with Proteinase K (PK), and 15 μg of protein was used for SDS-PAGE. Following PK treatment, denaturants (UA/DOC) were added, followed by DTT at a final concentration of 20 mM and incubation at 30 °C for 2 h. The samples were then cooled to room temperature, and IAA was added to a final concentration of 25 mM. The mixture was shaken at 600 rpm for 1 min and then incubated in the dark at room temperature for 30 min.

Ammonium bicarbonate buffer (50 mM) was added to reduce the UA/DOC concentration to below 1.5 M. Then, 2 μg of trypsin was added, and the samples were incubated at 37 °C for 16 h. After desalting and lyophilization, the samples were reconstituted in 0.1% formic acid. Peptide concentration was measured by OD280. Finally, 2 μg of peptides were mixed with an appropriate amount of iRT standard peptides for DIA mass spectrometry analysis.

### 2.11. Establishment of Lip-MS Analysis Method

Chromatographic separation was performed using a Vanquish Neo system (Thermo Fisher, Waltham, MA, USA) followed by Data-Independent Acquisition (DIA) on an Astral high-resolution mass spectrometer (Thermo Scientific, Waltham, MA, USA) operated in positive ion mode. The precursor ion range was set from 380 to 980 *m*/*z*. MS1 spectra were acquired at a 240,000 resolution (at 200 *m*/*z*) with a normalized AGC target of 500% and a maximum injection time of 5 ms. For DIA MS2 scans, a 2 *m*/*z* isolation window was applied, HCD collision energy was set to 25 eV, and a normalized AGC target of 500% with a 3 ms maximum injection time was used. DIA data were analyzed with Spectronaut software (https://biognosys.com/software/spectronaut/).

### 2.12. Cell Culture and Treatment

Rat synovial fibroblasts (RSFs) were cultured in complete DMEM medium (Gibco, China) supplemented with 5.5 mmol/L D-glucose, 10% fetal bovine serum (FBS, TransSerum, Australia), and 1% penicillin-streptomycin. Prior to experiments, cells were synchronized by serum starvation for 24 h under standard culture conditions (37 °C, 95% humidity, 5% CO_2_). The cells were then pretreated for 2 h with either sodium fluoride or the respective test compounds.

The experimental groups were designated as follows: Control, Sodium Fluoride (600 μM NaF), Quercetin (600 μM NaF + 5 μM Quercetin), Epicatechin (600 μM NaF + 20 μM Epicatechin), Emodin (600 μM NaF + 5 μM Emodin), and Ellagic Acid (600 μM NaF + 1 μM Ellagic acid).

Cell viability and proliferation were assessed using the MTT assay. Briefly, RSFs were seeded in 96-well plates at an initial density of 2.5–3 × 10^4^ cells per well, including blank control wells (without cells) and untreated control wells (cells with medium only). Each treatment group consisted of six replicate wells. Following 48 h of treatment with NaF (Guangrun Bio, Nanjing, China) at the specified concentrations, 10 μL of MTT reagent was added to each well and incubated for 4 h at 37 °C. Optical density at 490 nm was determined using a Varioskan™ LUX multimode microplate reader (Thermo Fisher Scientific, Waltham, MA, USA).

### 2.13. Real-Time Quantitative PCR (RT-qPCR) Analysis

Total RNA from RSFs was extracted using the RNeasy Total RNA Kit (Vazyme, Nanjing, China). Following genomic DNA removal, cDNA was generated using the EasyScript^®^ Genomic DNA Removal and cDNA Synthesis Kit (TransGen Biotech, Beijing, China). Quantitative real-time PCR was performed on the resulting cDNA with PerfectStart^®^ Green qPCR SuperMix (TransGen Biotech, Beijing, China) on a Bio-Rad CFX Manager 3.1 system (Bio-Rad Laboratories). Relative mRNA expression was calculated via the 2^(−ΔΔCt) method, using β-Actin as the reference gene. All primers were custom-synthesized by Sangon Biotech (Shanghai, China); the primer sequences are listed in Table 2.

### 2.14. Determination of Intracellular Reactive Oxygen Species (ROS) Levels

Intracellular reactive oxygen species (ROS) levels were measured using the fluorescent probe 2’,7’-dichlorofluorescin diacetate (DCFH-DA). After the indicated treatments, cells were incubated with 10 μM DCFH-DA in serum-free medium at 37 °C for 30 min in the dark. Following incubation, cells were washed three times with ice-cold phosphate-buffered saline (PBS) to remove excess probe. Fluorescence intensity was immediately analyzed using a NovoCyte flow cytometer (ACEA Biosciences, Hangzhou, China). Data acquisition and analysis were performed with NovoExpress software (https://www.agilent.com/product/novoexpress-software) (Agilent Technologies, Santa Clara, CA, USA). The mean fluorescence intensity (MFI) was calculated to quantify intracellular ROS levels, reflecting the degree of oxidative stress.

### 2.15. Detection of Inflammatory Cytokine Protein Expression (ELISA)

Cell culture supernatants were collected, and the secretion levels of inflammatory cytokines (TNF-α, IL-1β, IL-6) were quantitatively measured using commercial ELISA kits (R&D Systems, Fanyin, Shanghai, China) according to the manufacturer’s instructions. The changes in cytokine secretion across experimental groups were assessed to validate the corresponding mRNA expression results obtained from RT-qPCR.

### 2.16. Cell Cycle Analysis

Cell cycle distribution was analyzed by flow cytometry. After treatment, cells were harvested, washed with PBS, and fixed in 70% cold ethanol at 4 °C overnight. Fixed cells were washed twice with PBS and incubated with propidium iodide (PI) staining solution containing RNase A (Keygen Biotech, Jiangsu, China) at 37 °C for 30 min in the dark. Cell cycle profiles were determined using a NovoCyte flow cytometer (ACEA Biosciences, Hangzhou, China). Data were analyzed with NovoExpress software (Agilent Technologies, Santa Clara, CA, USA). The percentages of cells in the G0/G1, S, and G2/M phases were calculated to evaluate the effects of NaF and drug treatments on cell cycle progression and proliferation.

### 2.17. Immunofluorescence Staining and Analysis

RSFs were cultured on glass coverslips in 24-well plates at a density of 1 × 10^5^ cells per well for 24 h. After the designated treatments, cells were fixed with 4% paraformaldehyde (PFA) in PBS for 15 min at room temperature and washed three times with PBS. Cells were permeabilized using 0.2% Triton X-100 (Solarbio, Beijing, China) for 10 min and then blocked with 5% bovine serum albumin (BSA) in PBS for 1 h at room temperature to minimize non-specific binding.

Cells were treated with a 1:200 dilution of anti-p53 primary antibody in blocking solution and incubated overnight at 4 °C. Cells were washed three times with PBS and then incubated with a Cy3-conjugated secondary antibody for 1 h at room temperature in the dark. After washing, coverslips were mounted onto glass slides with an anti-fade medium containing 4′,6-diamidino-2-phenylindole (DAPI) to visualize nuclei.

Images were obtained using a Nikon Eclipse C1 fluorescence microscope (Nikon, Tokyo, Japan), and the fluorescence signal of p53 was quantified with ImageJ software (v1.52a, NIH, USA). For each coverslip, a minimum of five randomly selected fields were analyzed, with three independent experiments performed for statistical reliability.

### 2.18. Detection of Key Signaling Pathway Protein Expression by Western Blot

Total protein was isolated from cells using RIPA lysis buffer, and concentrations were assessed via BCA assay. Denaturation was performed by heating protein samples at 100 °C for 10 min in loading buffer. Equivalent amounts of protein were separated on 10% SDS-PAGE gels and subsequently transferred to 0.45 μm PVDF membranes for immunoblotting. Membranes were blocked with 5% skim milk at room temperature for 2 h and then incubated overnight at 4 °C with the following primary antibodies: anti-P53 (1:5000), anti-P-P53 (1:1000), anti-P21 (1:2000), and anti-α-tubulin (1:5000). The next day, secondary antibody incubation was carried out for 2 h at room temperature. Protein signals were detected using the NcmECL High Kit (NCM Biotech, Suzhou, China) and quantified via densitometric analysis with Image Lab software (Bio-Rad, Hercules, CA, USA, version 5.2).

### 2.19. Statistical Analysis

All algorithms and data analyses were performed using R software (version 4.3.2). Comparisons of continuous variables between two groups were conducted using Student’s *t*-test in GraphPad Prism (version 9.0), while comparisons among multiple groups were carried out using one-way ANOVA followed by Dunnett’s post hoc test. The significance levels were defined as follows: * *p* < 0.05, ** *p* < 0.01, *** *p* < 0.001 (indicating between-group differences); # *p* < 0.05, ## *p* < 0.01, ### *p* < 0.001 (denoting other specific comparisons).

## 3. Results

### 3.1. Integrated Bioinformatic Analysis Identifies Core Genes Associated with Fluoride-Related Osteoarthritis

**Figure 1:**
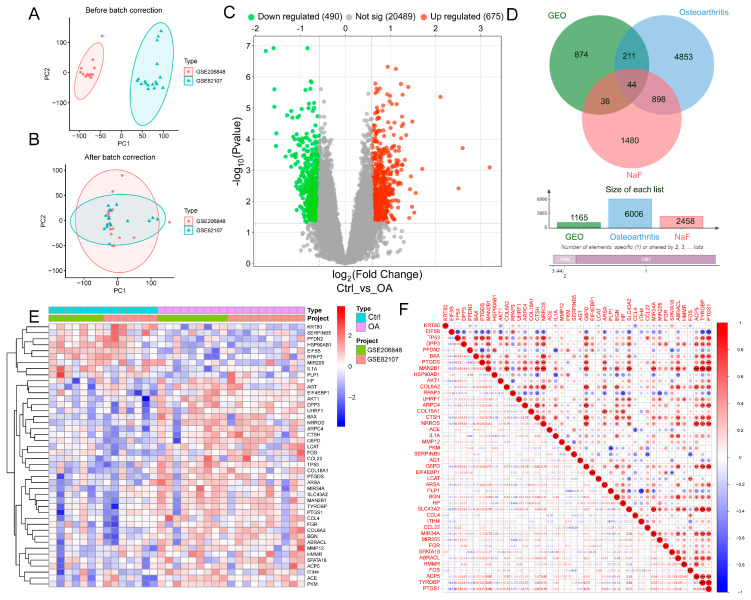
Bioinformatic identification of fluoride-associated hub genes in osteoarthritis through integrated GEO data analysis. (**A**,**B**) PCA results showing dimensionality reduction before and after dataset processing. (**C**) Volcano plot of differentially expressed genes (DEGs) in OA, with red indicating upregulated genes and green indicating downregulated genes. (**D**) Venn diagram showing the intersection of sodium fluoride-related genes, OA genes, and DEGs. (**E**) Heatmap of DEGs expression profiles. (**F**) Correlation analysis of co-associated genes. * *p* < 0.05, ** *p* < 0.01, *** *p* < 0.001 indicate statistical significance of the correlation coefficients.

We analyzed two microarray datasets, GSE82107 and GSE206848, to investigate the potential relationship between fluoride exposure and the development of OA. These datasets consisted of 14 healthy control samples and 19 OA samples. Initially, batch effects between the two datasets were identified and corrected using the “sva” (version 3.50.0) package in R (version 4.3.2). Principal Component Analysis (PCA) of the raw data revealed a clear separation between the two datasets before correction (Figure 1A). After the removal of batch effects, however, the samples from both groups were distributed homogeneously, indicating successful integration of the datasets for further analysis (Figure 1B). This confirmed that the combined dataset was reliable for subsequent analyses.

Comparative analysis between OA and control samples identified 1165 differentially expressed genes (DEGs), of which 675 were upregulated and 490 downregulated (Figure 1C). Further integration of these DEGs with gene sets from the GEO database, known OA-related genes, and NaF-associated genes allowed us to identify 44 overlapping candidate genes (Figure 1D). The heatmap demonstrated that OA and control groups exhibited markedly different expression patterns for these genes (Figure 1E), while correlation analysis revealed close functional interactions among these genes (Figure 1F). Consequently, a total of 44 core F-OA genes were ultimately identified, with their detailed information presented in Table 3.

### 3.2. Prediction of the Potential Mechanisms of RRJ Active Ingredients Against F-OA Based on Network Pharmacology Analysis

**Figure 2:**
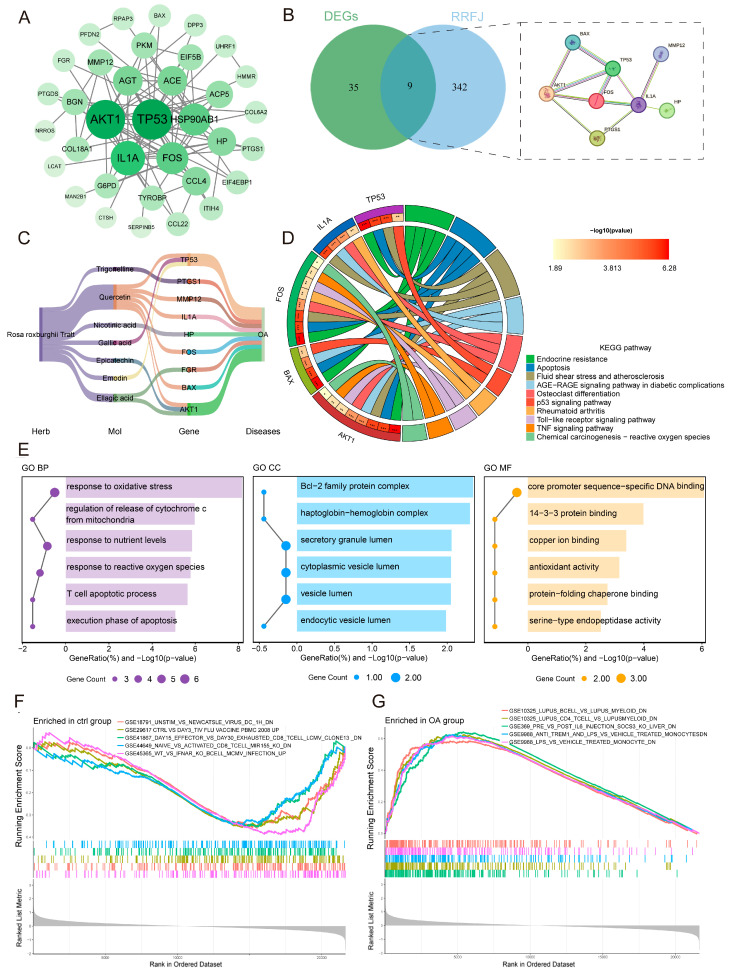
Integrated Bioinformatics Analysis of the Protective Mechanisms of RRJ. (**A**) PPI network of co-associated genes. (**B**) Venn diagram and PPI analysis of fluorosis-related OA co-associated genes. (**C**) Compound-Target-Disease Sankey diagram. (**D**) KEGG circos plot. * *p* < 0.05, ** *p* < 0.01, *** *p* < 0.001 indicate statistical significance of the gene-pathway associations. (**E**) Dual-axis bubble plot of GO enrichment. (**F**) Characteristic pathways of GSEA in healthy control group. (**G**) Characteristic pathways of GSEA in OA group.

To systematically elucidate the molecular mechanism of RRJ active components in the treatment of F-OA, we first constructed a protein-protein interaction (PPI) network using the 44 previously identified core genes through the STRING database (confidence score > 0.4; Figure 2A). Subsequently, based on the previously established RRJ bioactive ingredient database [17], we collected 351 potential targets associated with the 44 ingredients from SwissTargetPrediction and TCMSP and performed an intersection analysis with the core genes. The results showed that these overlapping targets occupied central hub positions in the PPI network (Figure 2B), including nine core targets such as TP53, PTGS1, MMP12, and IL1A. Further analysis revealed that seven of the 44 RRJ ingredients, including Quercetin, Epicatechin, Emodin, and Ellagic acid, were interconnected with these core targets, as depicted in Figure 2C.

Pathway enrichment analysis of these target genes indicated their significant enrichment in oxidative stress, the p53 signaling pathway, and the TNF signaling pathway (Figure 2D). Gene Ontology (GO) analysis indicated that these genes were primarily involved in biological processes such as response to oxidative stress, regulation of cytochrome c release from mitochondria, and T cell apoptotic processes, with molecular functions including DNA binding, antioxidant activity, and protein folding chaperone binding (Figure 2E). Gene Set Enrichment Analysis (GSEA) further demonstrated that gene sets from the control group were predominantly enriched in pathways related to mitochondrial energy metabolism, while the OA group showed significant enrichment in inflammation and stress response pathways (Figure 2F,G). In summary, integrating evidence from core gene identification, multi-faceted enrichment analyses, and the existing literature, we found that TP53 and its associated p53 signaling pathway play pivotal roles in the aforementioned processes of oxidative stress, inflammation, immune response, and apoptosis. The bioactive components related to RRJ may exert a systemic protective effect against F-OA by modulating multiple pathways, including oxidative stress, apoptosis, and inflammation, through the TP53/p53 signaling axis.

### 3.3. Molecular Docking Analysis of Active Ingredients Related to RRJ

Molecular docking was used to assess the interaction mechanisms between the principal bioactive constituents of RRJ and core target proteins. The binding energy, a crucial thermodynamic parameter, indicates the stability of protein–ligand complexes, with lower values corresponding to more stable binding. Energies below 0 kcal/mol signify a spontaneous interaction, whereas values below −5.0 kcal/mol are indicative of strong binding affinity.

**Figure 3:**
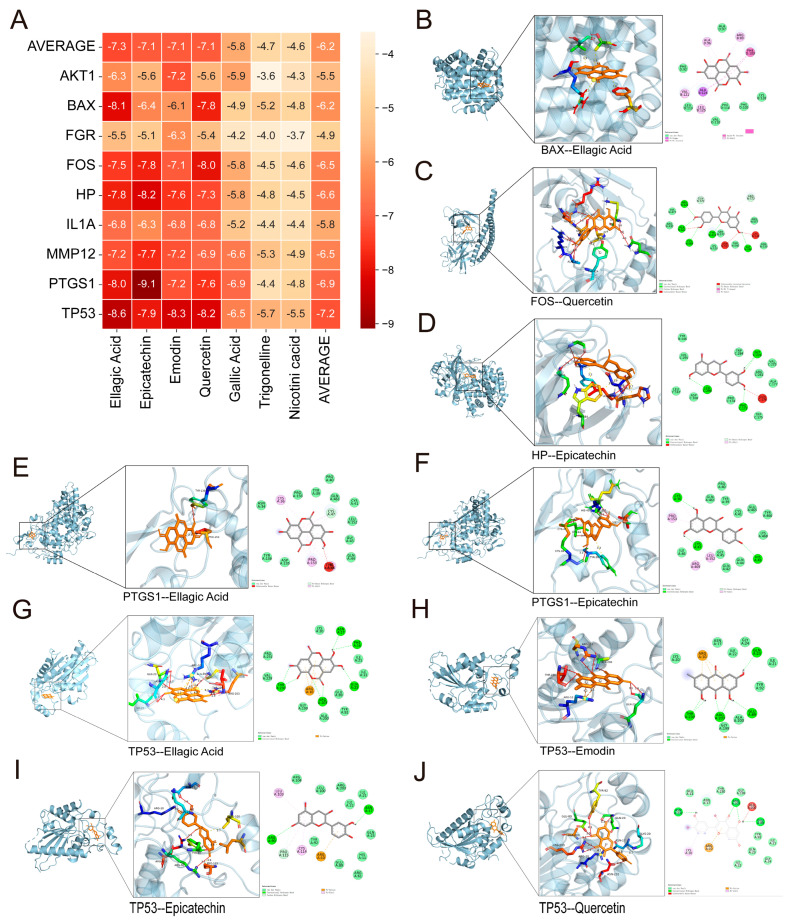
The 7 core active compounds of RRJ docked with 9 promising target proteins associated with fluoride-related OA. (**A**) Heat map showing the minimum binding energies between active compounds and targets. (**B**–**J**) Visualization of representative molecular docking models with binding energies lower than −9 kcal/mol, indicating strong binding affinity.

The molecular docking results revealed that the binding energies between the active components of RRJ and the core targets ranged from −5.0 to −9.0 kcal/mol (Figure 3A), reflecting potent and stable intermolecular interactions between the bioactive compounds and target proteins. Representative docking poses were also shown (Figure 3B–J), illustrating the binding modes and key interaction sites between the ligands and receptors. These results suggest that the active components of RRJ may exert their therapeutic effects by achieving high-affinity binding with disease-related target proteins, thereby modulating the pathological processes of F-OA. Finally, based on the average binding free energy from molecular docking, we further predicted four components—Quercetin, Epicatechin, Emodin, and Ellagic acid—to be the highly core target-associated bioactive ingredients in RRJ. These results suggest that these highly associated RRJ components may regulate the pathological process of fluoride-induced osteoarthritis through high-affinity binding to disease-relevant target proteins.

### 3.4. RRJ Highly Correlated Active Ingredients Can Restore the Viability and Function of NaF-Damaged Synovial Fibroblasts

**Figure 4:**
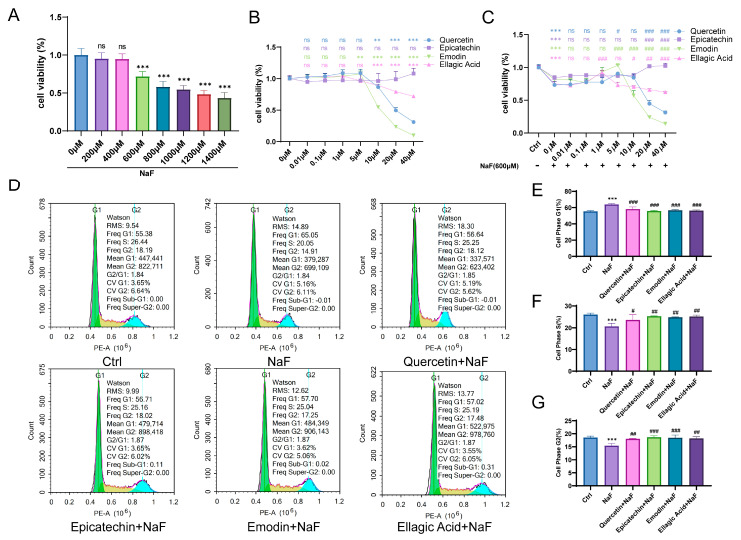
RRJ active components alleviate NaF-induced cytotoxicity and cell cycle arrest. (**A**) Cell viability after 48 h NaF treatment. (**B**,**C**) Viability rescue by polyphenols (600 µM NaF). (**D**) Representative flow cytometry histograms showing cell-cycle distribution under different treatments (Ctrl, NaF, Epicatechin + NaF, Emodin + NaF, Quercetin + NaF, and Ellagic Acid + NaF). (**E**) Quantification of the percentage of cells in the G1 phase. (**F**) Quantification of the percentage of cells in the G2/M phase. (**G**) Quantification of the percentage of cells in the S phase. Statistical significance was determined using one-way ANOVA followed by Tukey’s post-hoc test (** *p* < 0.01, *** *p* < 0.001 vs. control; # *p* < 0.05, ## *p* < 0.01, ### *p* < 0.001 vs. NaF-treated group; ns, not significant). Data are presented as mean ± SD (*n* = 3).

To elucidate the protective effects of the four highly RRJ-associated active components—Quercetin, Epicatechin, Emodin, and Ellagic acid—in alleviating fluoride-induced osteoarthritic cell damage, we first evaluated the cytotoxicity of sodium fluoride (NaF) on rat synovial fibroblasts. RRJ NaF caused a clear dose-dependent decline in cell viability, with viability reduced to below 50% at 1000 and 1400 μM (Figure 4A). We next assessed the intrinsic cytotoxicity of Quercetin, Epicatechin, Emodin, and Ellagic acid (Figure 4B) and established a cellular injury model using 600 μM NaF. Under this condition, all four active components significantly rescued NaF-induced loss of cell viability (Figure 4C). Accordingly, the following treatment regimens were applied: Sodium Fluoride (600 μM NaF), Quercetin (600 μM NaF + 5 μM Quercetin), Epicatechin (600 μM NaF + 20 μM Epicatechin), Emodin (600 μM NaF + 5 μM Emodin), and Ellagic Acid (600 μM NaF + 1 μM Ellagic acid). Because NaF-mediated cytotoxicity was accompanied by alterations in cell-cycle progression, we further examined cell-cycle distribution. NaF exposure induced pronounced G1-phase arrest, reflected by an elevated G1 fraction and reduced S and G2/M fractions (Figure 4D), whereas each active component effectively reversed this arrest and restored normal cell-cycle dynamics (Figure 4E–G).

**Figure 5:**
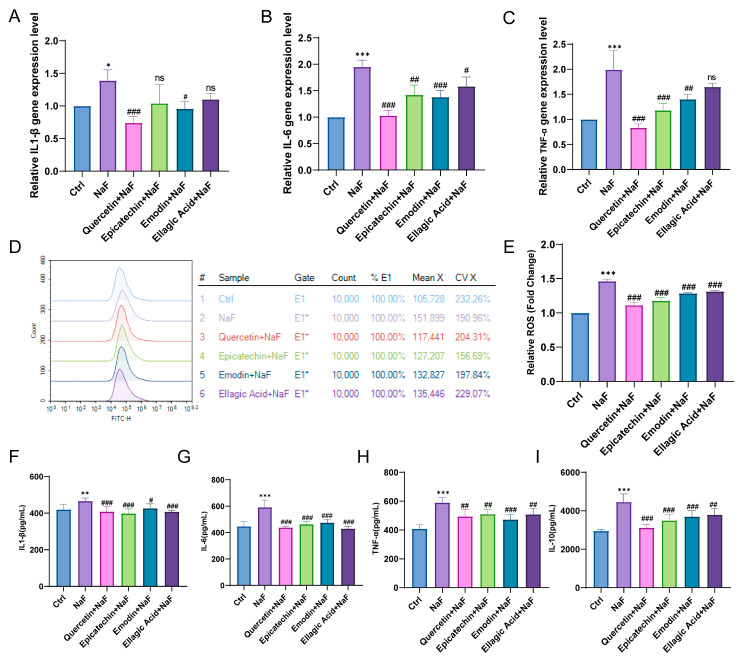
RRJ active components attenuate NaF-induced inflammatory responses, oxidative stress, and p53 activation. (**A**) Relative mRNA expression of IL-1β as determined by qPCR (*n* = 3). (**B**) Relative mRNA expression of IL-6 as determined by qPCR (*n* = 3). (**C**) Relative mRNA expression of TNF-α as determined by qPCR (*n* = 3). (**D**) Representative flow cytometry histograms of DCFH-DA-stained cells showing intracellular ROS levels. (**E**) Quantification of ROS production expressed as fold-change relative to control (*n* = 3). (**F**) IL-1β protein concentrations determined by commercial ELISA kit (*n* = 6). (**G**) IL-6 protein levels measured by ELISA (*n* = 6). (**H**) TNF-α protein levels assessed by ELISA (*n* = 6). (**I**) IL-10 protein levels assessed by ELISA (*n* = 6). Statistical significance was determined using one-way ANOVA followed by Tukey’s post-hoc test (* *p* < 0.05, ** *p* < 0.01, *** *p* < 0.001 vs. control; # *p* < 0.05, ## *p* < 0.01, ### *p* < 0.001 vs. NaF-treated group; ns, not significant). Data are presented as mean ± SD.

To further explore the anti-inflammatory and antioxidant mechanisms involved, we quantified the mRNA levels of inflammatory cytokines and ROS. qPCR analysis revealed that NaF markedly upregulated the mRNA expression levels of IL-1β, IL-6, and TNF-α, whereas all four active components of RRJ significantly counteracted this effect (Figure 5A–C). NaF also induced a substantial increase in ROS production, which was effectively scavenged by these active components (Figure 5D,E). At the protein level, NaF stimulated the secretion of IL-1β, IL-6, TNF-α and IL-10, and this induction was strongly inhibited by treatment with the active components (Figure 5F–I).

Collectively, these findings demonstrate that Quercetin, Epicatechin, Emodin, and Ellagic acid can counteract fluoride ion toxicity by enhancing cell viability, suppressing inflammatory responses, reducing oxidative stress, and restoring normal cell cycle regulation. These combined pharmacological effects suggest their potential in effectively alleviating fluoride poisoning-induced osteoarthritis.

### 3.5. Protein Conformational Remodeling and Functional Network Reprogramming Induced by Core Components of RRJ Identified Through LiP–MS

**Figure 6:**
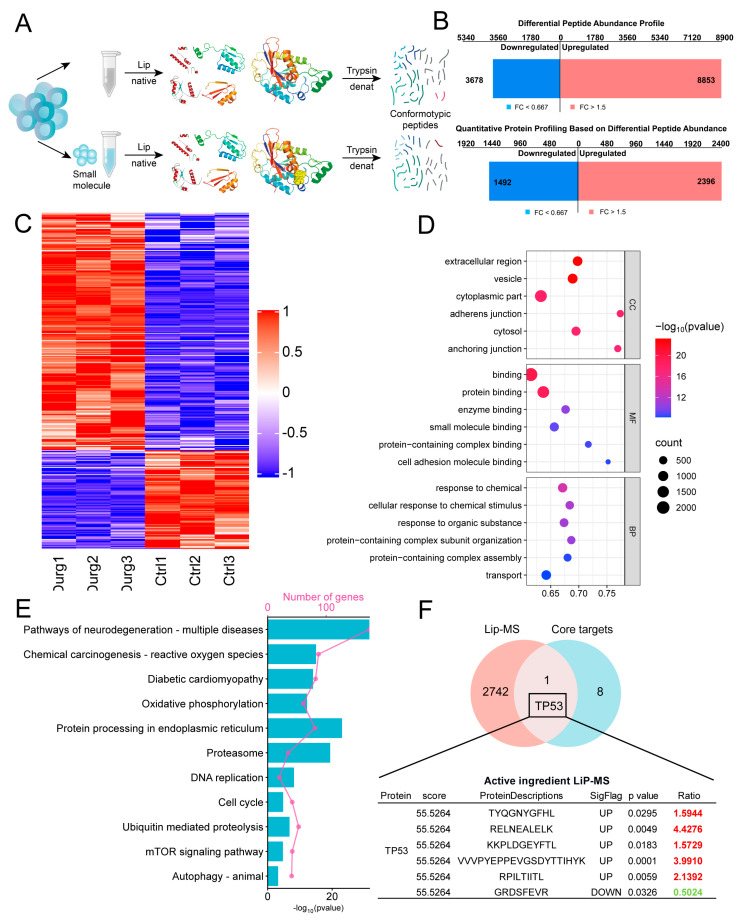
Protein conformational remodeling and network reprogramming by RRJ revealed by LiP–MS. (**A**) Schematic of the LiP–MS workflow. (**B**) Summary of differential peptides/proteins. (**C**) Clustering heatmap of altered proteins, showing clear segregation between groups. (**D**) GO enrichment analysis of altered proteins across cellular component, molecular function, and biological process categories. (**E**) KEGG pathway analysis. (**F**) Target integration.

To determine whether Quercetin, Epicatechin, Emodin, and Ellagic acid can induce protein conformational remodeling in synovial fibroblasts, we employed Limited Proteolysis–Mass Spectrometry (LiP–MS) to compare conformation-specific peptides between control and treated samples under both native and denatured conditions (Figure 6A). Quantitative analysis revealed significant changes in peptide abundance between the groups treated with Quercetin, Epicatechin, Emodin, and Ellagic acid and the control group. A total of 8853 peptides were significantly upregulated and 3678 were downregulated, corresponding to 2396 upregulated and 1492 downregulated proteins, respectively (Figure 6B). These results indicate that the highly RRJ-associated active components induced extensive alterations in protein structural states. Unsupervised clustering demonstrated that the treatment and control groups were clearly segregated, with significantly increased expression of proteins in the treated samples (Figure 6C). This distinct clustering pattern highlights the substantial remodeling of both protein conformation and expression profiles induced by the highly RRJ-associated active components.

Functional enrichment analysis of the differentially expressed proteins showed significant enrichment in extracellular regions, vesicles, cytoplasmic components, and adhesion junctions (Figure 6D). KEGG pathway analysis demonstrated significant enrichment in pathways related to neurodegenerative diseases and oxidative stress, including oxidative phosphorylation, diabetic cardiomyopathy, and endoplasmic reticulum protein processing (Figure 6E). These results indicate that the highly RRJ-associated active components regulate processes related to cellular homeostasis, stress response, immunity, and inflammation through extensive remodeling of protein conformations and expression networks. Notably, targeted fishing for the TP53 protein revealed consistency between the LiP-MS results and the predicted core target TP53, with multiple peptides showing significant upregulation after treatment. This suggests that these active components may directly enhance the structural stability of TP53, thereby perturbing the P53 pathway and exerting key regulatory effects at the molecular level.

### 3.6. Comprehensive MD and MMPBSA Analyses Reveal Stable and Favorable Binding of Highly Relevant RRJ Active Compounds to TP53

**Figure 7:**
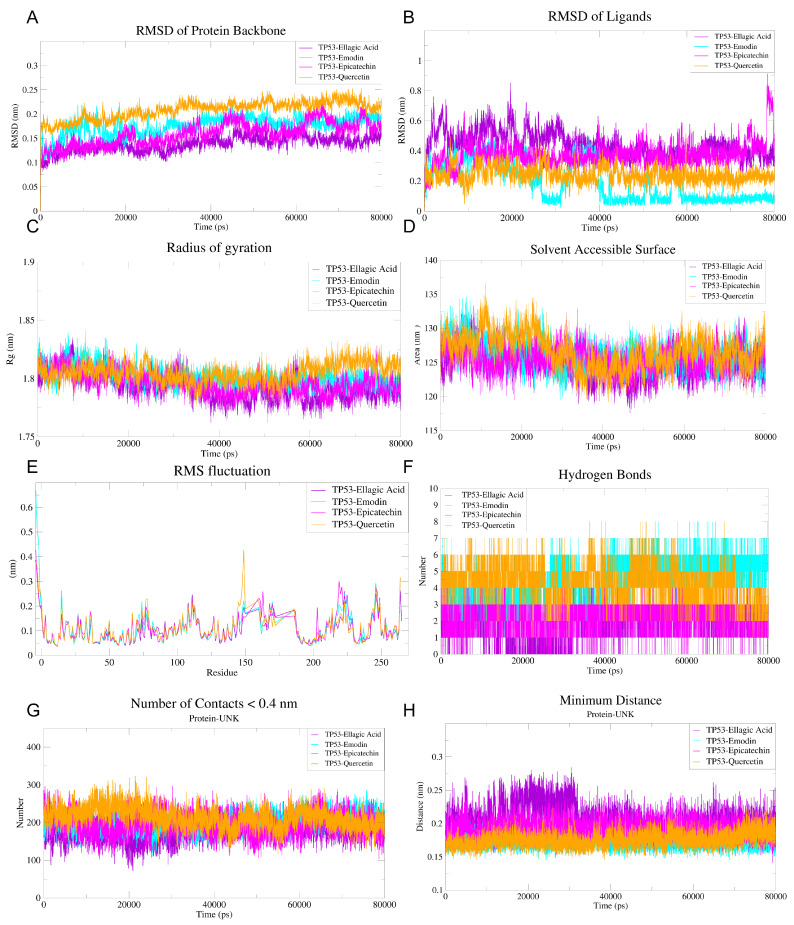
Molecular dynamics simulation analysis of TP53-ligand complexes. (**A**) RMSD (Root Mean Square Deviation) of the protein backbone over 80 ns of simulation time, indicating the stability of the protein structure in the presence of different ligands. (**B**) RMSD of the ligands over the same simulation period, showing the stability of the ligands when bound to the protein. (**C**) Radius of gyration (Rg) as a measure of the compactness of the protein structure during the simulation, reflecting the overall protein stability. (**D**) Solvent accessible surface area (SASA) of the protein over the simulation time, providing insights into the protein’s exposure to the solvent environment. (**E**) RMS fluctuation of the protein residues, highlighting the flexibility of different regions of the protein upon ligand binding. (**F**) Time course of ligand-TP53 hydrogen bonds (80 ns). (**G**) Number of atomic contacts (<0.4 nm) between ligands and TP53. (**H**) Minimum binding distance between ligands and TP53 over time.

To further elucidate the molecular mechanisms underlying the binding of Ellagic acid, Emodin, Epicatechin, and Quercetin to TP53, we performed 80 ns molecular dynamics (MD) simulations to systematically evaluate the conformational dynamics, structural stability, and interaction characteristics of the complexes. Global structural analysis showed that the backbone root mean square deviation (RMSD) of TP53 in all complexes stabilized between 0.15–0.30 nm, indicating overall structural stability, with the TP53–Quercetin complex exhibiting slightly higher conformational flexibility during the later stages of the simulation (Figure 7A). Ligand RMSD analysis revealed that Ellagic acid and epicatechin remained highly stable within the binding pocket (RMSD < 0.3 nm), whereas emodin and Quercetin displayed larger fluctuations (up to ~0.8 nm), suggesting potential conformational rearrangements or relatively weaker binding stability (Figure 7B).

Assessment of the radius of gyration (Rg) demonstrated that TP53 maintained a compact and stable structure upon ligand binding (Figure 7C). Solvent-accessible surface area (SASA) values exhibited a slight decreasing trend across all systems, while the TP53–Quercetin complex maintained relatively higher SASA, implying a more solvent-exposed binding conformation (Figure 7D). Per-residue root mean square fluctuation (RMSF) analysis indicated elevated flexibility in the N- and C-terminal regions of TP53. Notably, moderate fluctuations were observed near residues 150 and 250 in the TP53–Ellagic acid and TP53–Quercetin complexes, reflecting ligand-induced increases in local dynamic flexibility (Figure 7E).

To evaluate binding stability, we further analyzed the time-dependent evolution of hydrogen bonds, atomic contacts within 0.4 nm, and the minimum distance between TP53 and each ligand. The TP53–Ellagic acid complex maintained 1–4 hydrogen bonds throughout the simulation, with a marked increase after 40 ns, suggesting progressive stabilization (Figure 7F). The TP53–Emodin complex exhibited a consistently higher hydrogen-bond count (2–6), whereas the TP53–epicatechin complex fluctuated between 1–4 hydrogen bonds (Figure 7F). The TP53–Quercetin complex formed the most extensive hydrogen-bond network, reaching up to 7–8 interactions, highlighting its strong polar binding characteristics (Figure 7F).

Atomic contact analysis showed that the TP53–Quercetin and TP53–Emodin complexes displayed the highest numbers of close contacts (~250–350 atoms), indicative of compact binding interfaces, while TP53–Ellagic acid and TP53–Epicatechin formed fewer contacts (~150–250 atoms) (Figure 7G). The minimum distance between TP53 and all four ligands remained <0.3 nm, confirming tight binding throughout the simulation. Among these, the TP53–Ellagic acid complex showed larger fluctuations in minimum distance, implying possible binding-mode adjustments, whereas the TP53–Quercetin complex maintained a relatively stable minimum distance, further supporting its strong binding affinity (Figure 7H).

**Figure 8:**
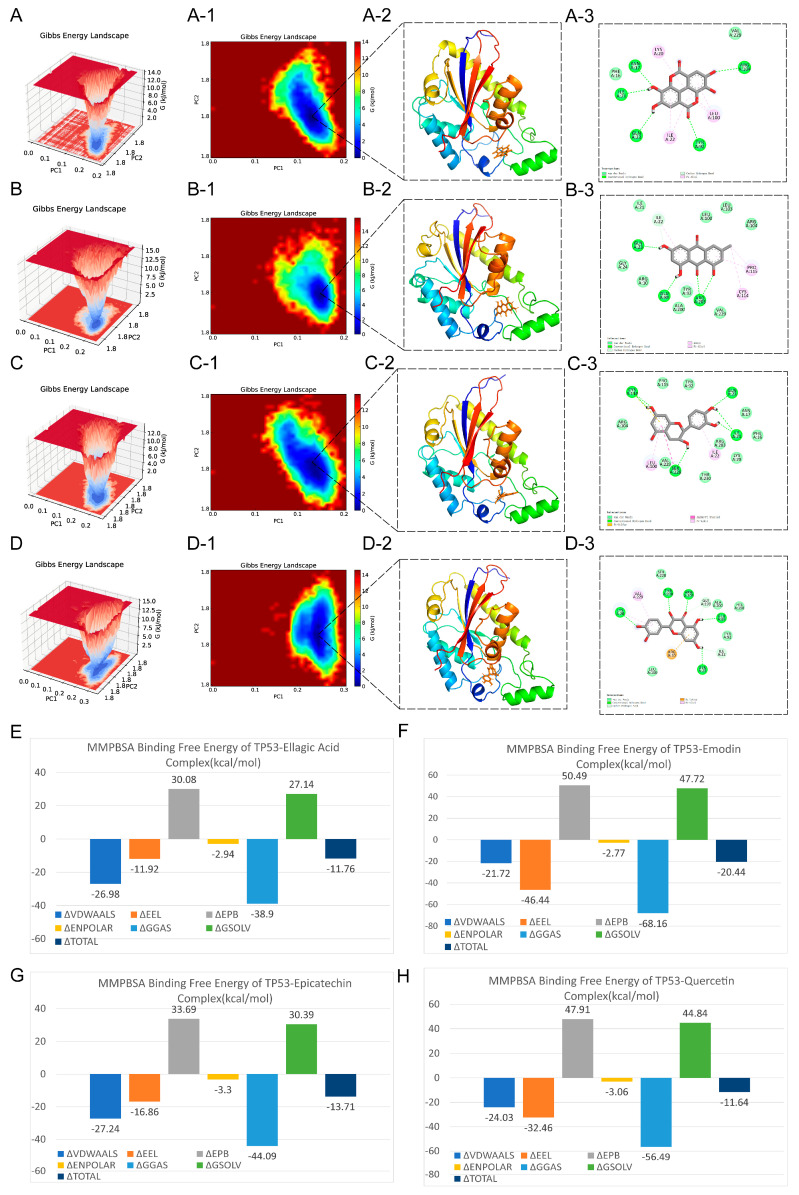
Gibbs free energy landscapes and binding energy decomposition of TP53-ligand complexes. (**A**–**D**) 3D GFE landscapes of TP53 with Ellagic acid (**A**), Emodin (**B**), Epicatechin (**C**), and Quercetin (**D**), showing conformational distribution and thermodynamic stability during MD simulations. Dark blue regions indicate energy minima representing thermodynamically favored conformations. (**A-1**–**D-1**) Corresponding 2D free energy maps with color gradients from blue/green (stable) to red (unstable/high energy). (**A-2**–**D-2**) Representative lowest-energy structures with TP53 backbone in rainbow colors and ligands in brown; all ligands bind within the common active pocket, interacting with the β-sheet core and nearby loops. (**A-3**–**D-3**) 2D interaction diagrams depicting ligand-TP53 active site contacts: green dashed lines for hydrogen bonds, pink for hydrophobic/van der Waals interactions, and purple for π-π stacking or electrostatic interactions. (**E**–**H**) show the energy component breakdowns of TP53 complexes with Ellagic acid (**E**), Emodin (**F**), Epicatechin (**G**), and Quercetin (**H**), respectively. Note: VDWAALS: van der Waals energy; EEL: electrostatic energy; EPB: electrostatic polar solvation energy; ENPOLAR: non-polar solvation energy; GGAS: gas-phase free energy; GSOLV: solvation free energy.

To assess thermodynamic stability and conformational diversity, Gibbs free-energy (GFE) landscapes were constructed using principal component analysis (PCA). Distinct low-energy basins were observed for all complexes (Figure 8A–D). The TP53–Ellagic acid complex exhibited the narrowest and deepest free-energy basin, reflecting high conformational stability. In contrast, the TP53–Emodin complex displayed a broader basin, suggesting the presence of multiple favorable low-energy states and greater conformational adaptability. The TP53–Epicatechin and TP53–Quercetin complexes showed basins of moderate depth and width, representing an intermediate balance between flexibility and stability.

We further quantified the binding energetics of TP53–ligand interactions using MMPBSA decomposition analysis (Figure 8E–H). All four ligands exhibited negative total binding free energies, indicating thermodynamically favorable binding: Emodin (ΔG_total = −20.44 kcal·mol^−1^), Ellagic acid (−11.76 kcal·mol^−1^), Epicatechin (−13.71 kcal·mol^−1^), and Quercetin (−11.64 kcal·mol^−1^). Gas-phase contributions—including van der Waals and electrostatic interactions—were the primary sources of favorable binding (ΔG_gas: Ellagic acid = −38.90, Emodin = −68.16, Epicatechin = −44.09, Quercetin = −56.49 kcal·mol^−1^). The polar solvation term (ΔEPB) provided an opposing, unfavorable contribution (30.08, 50.49, 33.69, and 47.91 kcal·mol^−1^, respectively), while nonpolar solvation energies contributed slightly favorable effects (≈−2.9 to −3.3 kcal·mol^−1^). Overall, the balance between strong gas-phase interactions and partially offsetting polar solvation energies led to consistently favorable total binding energies for all four complexes.

### 3.7. Highly Correlated Active Ingredients in RRJ Alleviate NaF-Induced p53 Activation by Inhibiting p53 Expression and Promoting Proteasomal Degradation

**Figure 9:**
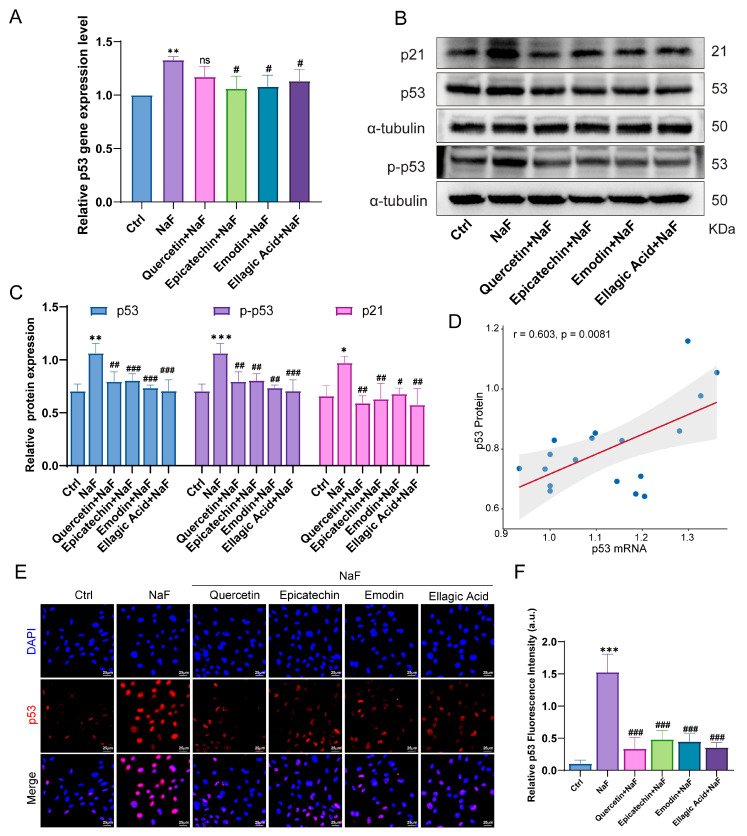
Effects of sodium fluoride and antioxidant compounds on p53 transcription, protein expression, and nuclear localization. (**A**) qPCR analysis of p53 mRNA levels (*n* = 3). (**B**,**C**) Western blot and quantification of p53 protein expression (*n* = 3). (**D**) Pearson correlation analysis was performed to evaluate the relationship between p53 mRNA levels and p53 protein abundance across all experimental groups. (**E**,**F**) Immunofluorescence detection of nuclear p53 accumulation (*n* = 5). Statistical significance was determined using one-way ANOVA followed by Tukey’s post-hoc test (* *p* < 0.05, ** *p* < 0.01, *** *p* < 0.001 vs. control; # *p* < 0.05, ## *p* < 0.01, ### *p* < 0.001 vs. NaF-treated group; ns, not significant). Data are presented as mean ± SD.

Finally, we evaluated the transcriptional and translational regulation of p53 following NaF exposure and examined the modulatory effects of the highly relevant active compounds in RRJ (Quercetin, Epicatechin, Emodin, and Ellagic acid). qPCR analysis showed that NaF markedly increased p53 mRNA expression, whereas all four compounds significantly suppressed this transcriptional upregulation (Figure 9A). Western blot results further confirmed that NaF substantially elevated p53 protein levels, while treatment with any of the four active components significantly attenuated this increase (Figure 9B,C). To determine whether NaF-induced p53 accumulation is transcriptionally driven, Pearson correlation analysis was performed between p53 mRNA and protein levels. A significant positive correlation was observed (r = 0.603, *p* = 0.0081; Figure 9D), indicating that NaF-induced p53 upregulation is partly transcriptionally mediated, with RRJ-derived compounds attenuating p53 activation through coordinated mRNA and protein suppression. Consistently, immunofluorescence showed pronounced nuclear p53 accumulation in NaF-treated cells, which was attenuated by antioxidant intervention (Figure 9E,F).

**Figure 10:**
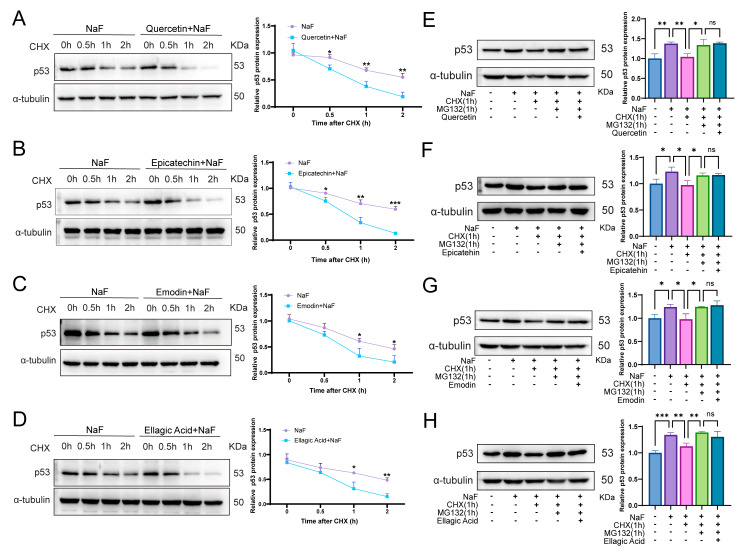
Effects of sodium fluoride and antioxidant compounds on p53 protein stability and proteasome-mediated degradation. (**A**–**D**) CHX chase assay assessing the kinetics of p53 degradation under NaF treatment in the presence of (**A**) Quercetin, (**B**) Epicatechin, (**C**) Emodin, and (**D**) Ellagic acid. (**E**–**H**) Evaluation of proteasome-dependent p53 degradation (CHX + MG132 assay) in cells co-treated with NaF and (**E**) Quercetin, (**F**) Epicatechin, (**G**) Emodin, and (**H**) Ellagic acid. Statistical significance was determined using one-way ANOVA followed by Tukey’s post-hoc test (* *p* < 0.05, ** *p* < 0.01, *** *p* < 0.001 vs. control; ns, not significant). Data are presented as mean ± SD (*n* = 3).

To further elucidate the mechanism underlying NaF-induced p53 accumulation, we performed CHX chase and CHX+MG132 inhibition assays to assess p53 protein stability and proteasome-dependent degradation. The CHX chase experiments demonstrated that NaF markedly extended the half-life of p53, indicating impaired degradation (Figure 10A–D), whereas Quercetin, Epicatechin, Emodin, and Ellagic acid restored the normal degradation rate and reversed NaF-induced p53 stabilization. Moreover, results from the CHX+MG132 assays showed that NaF inhibited proteasome-mediated p53 degradation, while the highly relevant active compounds in RRJ effectively reinstated this degradation process (Figure 10E–H).

Collectively, these findings demonstrate that NaF induces abnormal p53 accumulation by simultaneously enhancing its transcription and suppressing its proteasome-dependent degradation. In contrast, the highly correlated active ingredients in RRJ effectively counteract this pathological process through a dual regulatory mechanism, thereby protecting cells from fluoride-induced injury.

### 3.8. Qualitative and Quantitative Analysis of Four Active Ingredients in RRJ

**Figure 11:**
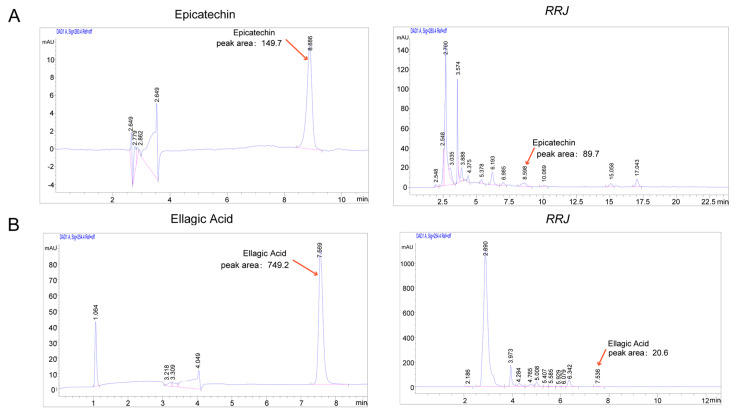
Quantitative HPLC analysis of epicatechin and ellagic acid in RRJ. (**A**) Representative HPLC chromatograms of Epicatechin. The left panel shows the chromatogram of the authentic reference standard, while the right panel displays the corresponding peak detected in the RRJ sample. (**B**) Representative HPLC chromatograms of Ellagic acid. The left panel shows the standard compound and the right panel depicts its corresponding peak in the RRJ sample. Both compounds were identified by comparison with authentic standards under identical chromatographic conditions.

Based on the integrated mechanistic analyses described above, four RRJ constituents showing the highest correlation with fluoride-responsive targets—Quercetin, Emodin, Epicatechin, and Ellagic acid—were subjected to qualitative and quantitative characterization. Authentic reference standards were employed for compound annotation. Quantitative HPLC analysis confirmed the presence of epicatechin and Ellagic acid in RRJ. Method validation results demonstrated excellent linearity for both epicatechin and ellagic acid, with correlation coefficients (R^2^) greater than 0.999. The LOD and LOQ values indicated high sensitivity of the method. Intra-day precision (RSD%) was 2.26% for epicate-chin and 8.31% for ellagic acid, while inter-day precision was below 10% for both compounds. The average recoveries ranged from 97% to 103%, indicating satisfactory accuracy. Detailed validation parameters are summarized in Supplementary Table S3. Finally, the concentrations of Epicatechin and Ellagic acid were measured to be 41.11 mg/mL and 0.63 mg/mL, respectively (Figure 11). In contrast, Quercetin and Emodin were not directly detected.

Furthermore, qualitative analysis was conducted on Epicatechin, Ellagic acid, as well as Quercetin and Emodin. RRJ was analyzed using ultra-high-performance liquid chromatography coupled with high-resolution mass spectrometry (UHPLC–HRMS) in both positive and negative ionization modes. A total of 130 chemical constituents were identified in RRJ. Epicatechin and Ellagic acid were consistently annotated in the UHPLC–HRMS dataset; qualitative analysis confirmed the presence of Epicatechin and Ellagic. In the qualitative detection, Quercetin and Emodin were not directly detected, and a derivative of Quercetin, namely, Quercetin 3,4′-diglucoside, was detected. Its glycosylated derivative is likely to be metabolized by liver enzymes to form free quercetin. It is highly likely that quercetin exists in the blood when the RRJ is absorbed by the human body, as detailed in Table A1 (Appendix A).

Collectively, these data establish epicatechin and Ellagic acid as the predominant quantifiable constituents among the highly correlated RRJ compounds, while Quercetin- and Emodin-associated species were present mainly in conjugated forms, providing a chemical basis for subsequent functional and mechanistic investigations.

## 4. Discussion

Our study demonstrates that polyphenolic constituents of Rosa roxburghii juice (RRJ) confer significant protection against fluoride-induced osteoarthritic damage through modulating oxidative stress, inflammatory pathways, and p53 proteostasis. These findings provide mechanistic insights into the therapeutic potential of medicine–food homology agents for managing fluoride-associated osteoarthritis (OA).

Integrated transcriptomic profiling identified TP53, FOS, BAX, and AKT1 as key fluoride-responsive hub genes, consistent with previous reports that implicate p53 activation in chondrocyte senescence, mitochondrial dysfunction, and cartilage degeneration in OA [25]. This convergence highlights p53 as a central stress response mediator in both fluoride toxicity and cartilage pathology.

Computational docking analyses further revealed that Quercetin, Epicatechin, Emodin, and Ellagic acid form stable interactions with the p53 protein, with Emodin exhibiting the strongest predicted binding affinity, corroborating structural studies indicating that natural polyphenols can directly modulate p53 conformation and stability [26]. In parallel, limited proteolysis mass spectrometry (LiP–MS) demonstrated widespread protein structural remodeling following RRJ treatment, particularly in pathways related to oxidative phosphorylation and proteostasis, consistent with the sensitivity of LiP–MS to capture ligand-induced conformational dynamics [27].

Functionally, sodium fluoride (NaF) exposure induced ROS accumulation, G1 phase arrest, and cytotoxicity in rat synovial fibroblasts, corroborating earlier evidence of fluoride-induced oxidative injury and mitochondrial damage [28,29,30,31].

Mechanistically, NaF exposure stabilized p53 by impairing its proteasomal degra-dation, a finding consistent with the literature, which suggests that fluoride disrupts proteostasis and cellular stress responses [32,33,34]

Several limitations of the present study should be acknowledged. First, the in vitro model employed does not fully recapitulate the biomechanical loading, immune interactions, and multicellular complexity characteristic of in vivo osteoarthritic tissues. Second, only a subset of LiP–MS-predicted targets was experimentally validated, and broader target confirmation is required in future investigations. Although in silico modeling suggested potential direct interactions between RRJ-derived polyphenols and p53, definitive structural validation, including biophysical binding assays and site-directed mutagenesis, remains necessary.

In addition, it should be noted that Quercetin and Emodin were not detected in the final quantitative analyses of RRJ, nor were they identified as free aglycones in UHPLC–HRMS profiling. This absence does not necessarily indicate that these constituents are precluded from their presence in RRJ, as differences in juice processing, sample preparation, or compound stability may influence their detectability. Accordingly, further targeted analyses and optimized extraction strategies are warranted to clarify the presence, forms, and bioavailability of Quercetin- and Emodin-related constituents in RRJ. Future studies should validate these findings using rodent models of fluoride-induced OA and employ structural proteomics to delineate specific polyphenol–protein interactions. Pharmacokinetic profiling will also be essential to determine which bioactive compounds most effectively accumulate in joint tissues. Collectively, our results support the promise of multi-component natural products such as RRJ as safe and multi-target therapeutic agents against fluoride-associated OA.

## 5. Conclusions

This study systematically elucidates the mechanistic basis by which RRJ mitigates F-OA, highlighting Epicatechin and Ellagic acid as key correlated constituents. Integrated transcriptomic, proteomic, and biochemical analyses identified TP53 as a potential core hub gene in F-OA pathogenesis. RRJ-associated constituents exhibited high predicted affinity toward TP53/p53 and induced protein conformational remodeling. Functionally, these compounds attenuated fluoride toxicity by suppressing p53 transcription and promoting proteasome-mediated p53 degradation, thereby limiting aberrant p53 activation.

Collectively, these findings provide a mechanistic rationale for the development of RRJ as a functional food for the prevention and intervention of fluoride-associated skeletal disorders and underscore the multi-target therapeutic potential of medicine–food homology substances in chronic toxicological diseases.

## Figures and Tables

**Table 1:** Gradient elution program for chemical composition data collection of RRJ.

Time (min)	Mobile Phase A (%)	Mobile Phase B (%)
Initial	95	5
3.0	75	25
8.5	55	45
14.0	5	95
17.0	2	98
17.2	95	5
20.0	95	5

**Table 2:** Primer sequences used for quantitative real-time PCR.

Gene Name	Primer Sequence (5′–3′)
TNF-α	F:AACACACGAGACGCTGAAGT R:TCCAGTGAGTTCCGAAAGCC
IL-1β	F:AGCTTCAGGAAGGCAGTGTC R:ATCCCACGAGTCACAGAGGA
IL-6	F:GCAAGAGACTTCCAGCCAGT R:GTCTCCTCTCCGGACTTGTG
P53	F:GTTCGTGTTTGTGCCTGTCC R:GTGCTCTCTTTGCACTCCCT
β-Actin	F:TGTCACCAACTGGGACGATA R:GGGGTGTTGAAGGTCTCAAA

**Table 3:** The list of 44 core F-OA genes identified in this study.

NO.	Gene	NO.	Gene
1	KRT80	23	SERPINB5
2	PTGDS	24	PKM
3	EIF5B	25	EIF4EBP1
4	MAN2B1	26	LCAT
5	DPP3	27	PLP1
6	BAX	28	ARSA
7	TP53	29	HP
8	AKT1	30	CCL4
9	COL6A2	31	BGN
10	PFDN2	32	SLC43A2
11	HSP90AB1	33	ITIH4
12	UHRF1	34	CCL22
13	RPAP3	35	MIR34A
14	CTSH	36	MIR205
15	NRROS	37	FGR
16	ARPC4	38	SPATA18
17	MMP12	39	ABRACL
18	G6PD	40	FOS
19	ACE	41	ACP5
20	COL18A1	42	HMMR
21	AGT	43	TYROBP
22	IL1A	44	PTGS1

## Data Availability

The original contributions presented in this study are included in the article/Supplementary Material. Further inquiries can be directed to the corresponding author(s).

## Supplementary Materials

The following supporting information can be downloaded at: https://www.mdpi.com/article/10.3390/antiox15030309/s1, Figure S1. Representative photograph of fresh fruits of Rosa roxburghii Tratt. cultivar ‘Guinong 5’ used in this study. Figure S2. Validation of the HPLC-DAD method for epicatechin and ellagic acid. (A) Calibration curve of epicatechin showing the linear relationship between peak area and concentration. (B) Calibration curve of ellagic acid showing the linear relationship between peak area and concentration. (C) Determination of the limit of detection (LOD) for epicatechin based on a signal-to-noise (S/N) ratio of 3. (D) Determination of the limit of quantification (LOQ) for epicatechin based on a signal-to-noise (S/N) ratio of 10. (E) Determination of the limit of detection (LOD) for ellagic acid based on a signal-to-noise (S/N) ratio of 3. (F) Determination of the limit of quantification (LOQ) for ellagic acid based on a signal-to-noise (S/N) ratio of 10. All experiments were performed in triplicate. Calibration curves were constructed using six concentration levels, and linear regression analysis was applied to calculate correlation coefficients (R^2^).

## Appendix A

**Table A1:** Chemical constituents identified in Rosa roxburghii juice (RRJ) by UHPLC-HRMS analysis.

ID	*m*/*z*	RT min	Formula	ppm	Compound Name	Product Ion
M118T833	118.0852	13.88	C5H11NO2	1.6	Sarcosine ethyl ester	[118.07, 118.09, 90.04, 119.07, 91.05]
M166T119_1	166.0845	1.98	C14H20N2O3	0.3	Valylphenylalanine	[120.08, 103.05, 131.05, 166.09, 93.07]
M124T1098	124.085	18.29	C11H16BrN5	1.8	[1-[(4-Bromo-3,5-dimethylpyrazol-1-yl)methyl]-3,5-dimethylpyrazol-4-yl]amine	[124.09, 107.06, 125.09, 124.11, 125.07]
M124T67	124.0379	1.11	C6H5NO2	1	Nicotinic acid	[124.04, 124.06, 96.04, 125.04, 124.02]
M291T193_3	291.0826	3.22	C15H14O6	0.9	Catechin	[139.04, 123.04, 147.04, 165.05, 291.09]
M279T822_3	279.1557	13.71	C16H22O4	0.9	Butyl terephthalate	[149.02, 121.03, 150.03, 205.09, 149.05]
M102T60	102.127	1	C6H15N	3.1	2-Ethylbutylamine	[102.13, 102.14, 103.13, 102.14, 102.11]
M149T837	149.022	13.95	C12H8O3S	0.3	2-(2-Thenoyl)benzoic acid	[149.02, 121.03, 121.04, 93.03, 111.04]
M409T227	409.1786	3.79	C20H28N2O5S	1.7	(-)-Tamsulosin	[409.18, 410.18, 102.29, 102.3, 409.31]
M114T1081	114.0902	18.02	C6H12FNO	2.5	4-Piperidinemethanol, 4-fluoro-	[114.09, 114.1, 96.08, 97.07, 114.06]
M122T109	122.0954	1.81	C15H16N2O	1.5	Carbanilide, 3,5-dimethyl-	[122.1, 107.07, 123.06, 123.04, 122.06]
M116T65	116.0702	1.08	C6H11NO4	1.8	N-Methyl-L-glutamic acid	[116.07, 116.14, 116.11, 98.06, 116.04]
M134T276	134.0593	4.61	C19H25NO10	1	1H-Indol-3-yl 6-O-((2R,3R,4R)-3,4-dihydroxy-4-(hydroxymethyl)tetrahydrofuran-2-yl)-.beta.-D-glucopyranoside	[134.06, 106.07, 135.08, 105.03, 106.03]
M128T253	128.1058	4.22	C7H13NO	0.9	8-Azabicyclo [3.2.1]octan-3-ol	[128.11, 129.02, 128.04, 128.07, 110.03]
M146T122	146.0585	2.04	C9H9NO2	0.7	3-Hydroxy-3-methyloxindole	[146.06, 128.05, 118.07, 91.05, 147.06]
M147T200	147.0425	3.33	C9H8O3	0.4	trans-3-Coumaric acid	[119.05, 147.04, 91.05, 148.05, 120.05]
M135T365	135.0791	6.09	C9H10O	0.8	p-Acetyltoluene	[135.08, 91.05, 105.07, 107.09, 136.02]
M139T209	139.0376	3.49	C7H6O3	0	3,4-Dihydroxybenzaldehyde	[139.04, 111.04, 93.03, 140.04, 95.05]
M151T306	151.1102	5.1	C10H16O2	0.7	(S)-8-Hydroxy-p-menth-1-en-6-one	[151.11, 109.07, 105.07, 109.1, 107.09]
M261T67	261.0915	1.12	C18H14O3	2.2	Dihydroisotanshinone I	[261.09, 261.15, 261.06, 262.1, 262.13]
M139T192	139.0366	3.21	C15H14O6	0.2	Epicatechin	[139.04, 111.04, 93.03, 95.05, 140.04]
M124T897	124.086	14.96	C14H17N5O	1.7	N-(4,6-Dimethyl-2-pyrimidinyl)-N’-(4-methoxyphenyl)guanidine	[124.09, 125.07, 124.04, 124.09, 124.08]
M121T822	121.0272	13.71	C7H7NO3	1.6	N,3-Dihydroxybenzamide	[121.03, 121.04, 93.03, 93.07, 111.04]
M130T53_2	130.085	0.89	C6H11NO2	0.2	N-Methyl-L-proline	[130.09, 130.05, 131.09, 130.07]
M161T268	161.0944	4.47	C18H28O10	0.1	1-O-((2E,4E)-9-Carboxy-8-hydroxy-2,7-dimethylnona-2,4-dienoyl)-.beta.-D-glucopyranose	[161.1, 143.09, 105.07, 128.06, 133.1]
M114T997	114.0905	16.62	C13H19FN2O	2.5	(2-Fluorobenzyl)-(2-morpholinoethyl)amine	[114.09, 114.1, 96.08, 97.07, 114.07]
M525T281	525.225	4.68	C28H35FO7	2.5	Amcinonide	[525.22, 526.23, 131.3, 393.18, 525.41]
M445T135	445.1275	2.25	C28H24O9	2.5	D-Ribofuranose, 1-acetate 2,3,5-tribenzoate	[445.13, 317.08, 446.13, 313.09, 417.13]
M131T67_2	131.0326	1.11	C5H6O4	0.3	Citraconic acid	[113.02, 131.03, 103.04, 101.02, 131.09]
M141T147	141.0522	2.45	C8H8O5	0.2	3-Methoxygallic acid	[141.05, 126.03, 109.03, 142.09, 98.04]
M147T41	147.1115	0.68	C6H14N2O2	0.1	Lysine	[130.09, 147.11, 129.1, 130.08]
M425T858	425.2098	14.3	C24H31FO4	1.3	(S)-AL 8810	[425.21, 365.19, 426.22, 425.08, 281.13]
M286T51	286.0866	0.85	C11H15N3O4S	3.7	1-(4-Mesyl-3-nitrophenyl)piperazine	[286.09, 166.05, 124.04, 150.01, 143.03]
M465T291_1	465.0976	4.85	C27H30O17	0.2	Quercetin 3,4′-diglucoside	[303.05, 465.15, 229.05, 304.05, 153.02]
M467T258	467.2233	4.31	C20H30N6O7	3.4	Glu-Tyr-Arg	[467.22, 449.21, 468.23, 467.1, 116.8]
M173T527	173.1306	8.78	C21H28O5	0.2	4-(Hydroxymethyl)-4,6a,9,12b-tetramethyl-4a,6,6a,12,12a,12b-hexahydro-4H,9H-benzo[f]pyrano [2,3-b]chromene-1,11(5H,10H)-dione	[173.13, 131.09, 158.11, 145.1, 143.09]
M391T906	391.2793	15.11	C24H38O4	0.6	Dioctyl phthalate	[149.02, 167.03, 121.03, 150.03, 149]
M743T875	743.3778	14.59	C35H60O15	3.8	3-O-(3-Methylbutanoyl)hex-2-ulofuranosyl 2-O-decanoyl-3,4-bis-O-(2-methylpropanoyl)hexopyranoside	[743.38, 744.38, 185.83, 743.69, 743.07]
M195T460	195.0632	7.67	C10H10O4	0.6	Caffeic acid methylester	[163.04, 135.04, 133.03, 93.07, 135.12]
M129T138_1	129.0531	2.29	C6H10O4	0.9	Monomethyl glutarate	[129.05, 101.06, 129.02, 111.04, 101.05]
M655T878	655.3264	14.64	C38H48O8	4.2	TETRAHYDROGAMBOGIC ACID	[655.33, 656.33, 163.82, 120.08, 163.84]
M161T561	161.1308	9.35	C15H24O	0	(-)-Caryophyllene oxide	[161.13, 119.09, 105.07, 133.1, 161.1]
M275T81_2	275.107	1.35	C19H14O2	1.1	7,8-Benzoflavanone	[275.11, 276.14, 230.14, 95.01, 258.13]
M156T46	156.0406	0.77	C5H5N3O3	0.6	Cytosine-5-carboxylic acid	[156.04, 112.05, 130.06, 110.07, 156.07]
M137T335	137.0947	5.58	C11H17NO2S	0.3	N-Mesitylethanamine	[137.1, 122.07, 109.1, 119.09, 107.05]
M116T286	116.106	4.77	C6H13NO	2	Methyl(oxolan-2-ylmethyl)amine	[116.11, 116.07, 117.07, 115.05, 116.14]
M125T131	125.0698	2.18	C7H8F2N2O	2	[6-(Difluoromethoxy)-3-pyridyl]methylamine	[125.07, 96.07, 95.06, 97.03, 125.02]
M109T324_1	109.064	5.4	C7H8O	2.4	3-Methylphenol	[109.07, 109.1, 91.05, 109.03, 113.96]
M123T72	123.0415	1.21	C7H6O2	1.1	Salicylaldehyde	[123.06, 95.05, 123.04, 124.04, 96.04]
M105T252	105.0327	4.2	C17H15N3OS	3.4	N-(5-Phenethyl-[1,3,4]thiadiazol-2-yl)benzamide	[105.03, 95.05, 105.07, 105.04, 103.05]
M132T79_2	132.1005	1.31	C6H15NO3	0.1	Triethylolamine	[132.1, 135.3, 101.96, 99.1, 93.37]
M118T118	118.0641	1.97	C10H9NO	1.6	1-(1H-Indol-5-yl)ethanone	[118.07, 118.09, 91.05, 90.04, 119.07]
M666T882	666.4269	14.71	C33H64NO10P	0.1	PAz-PC	[184.07, 125, 666.43, 98.98, 104.11]
M163T192	163.0371	3.19	C15H12O5	0.6	Butein	[163.04, 135.04, 117.03, 145.03, 107.05]
M105T323	105.069	5.39	C16H16ClNO2	2.9	2-(4-Chlorophenoxy)-N-(2-phenylethyl)acetamide	[105.07, 103.05, 95.05, 106.07, 105.04]
M109T31_2	109.1004	0.52	C14H26O	2.2	3-Oxabicyclo [10.3.0]pentadecane	[109.1, 113.96, 109.07, 109.08, 107.09]
M189T227	189.1254	3.79	C13H20O3	0.1	4-(1-Hydroxy-4-keto-2,6,6-trimethyl-2-cyclohexen-1-yl)-3-buten-2-ol	[189.13, 171.12, 119.09, 174.1, 161.13]
M133T202	133.0634	3.37	C12H14N2O3	0.4	4-Hydroxymephenytoin	[133.06, 105.07, 133.1, 134.06, 91.05]
M181T567	181.1204	9.45	C11H20O4	0.6	Undecanedioic acid	[181.12, 135.12, 163.11, 107.09, 121.1]
M119T350	119.0851	5.83	C10H12O2	1.9	1-(4-Methoxyphenyl)acetone	[91.05, 119.09, 119.05, 117.07, 119.07]
M105T389	105.0697	6.48	C12H13N3OS2	3.5	N-{5-[(4-Methylbenzyl)sulfanyl]-1,3,4-thiadiazol-2-yl}acetamide	[105.07, 103.05, 95.05, 106.07, 105.04]
M191T420	191.141	7.01	C13H22O3	0.1	1,2,3-Cyclohexanetriol, 5-(1,3-heptadien-1-yl)-	[191.14, 176.12, 145.1, 107.09, 105.07]
M187T59	187.0556	0.98	C6H12O5	1.4	2-Deoxy-D-glucose	[187.06, 187.11, 141.1, 128.07, 170.08]
M579T175	579.1433	2.91	C30H26O12	1.1	Procyanidin B2	[127.04, 139.04, 123.04, 163.04, 409.09]
M173T400_2	173.0943	6.66	C20H28O2	0.4	Retinal, 4-hydroxy-	[173.1, 145.1, 105.07, 158.07, 130.08]
M302T53	302.1176	0.88	C19H22FNO4	4	Diethyl 4-(4-fluorophenyl)-2,6-dimethyl-1,4-dihydropyridine-3,5-dicarboxylate	[302.12, 182.08, 143.03, 266.1, 284.11]
M321T52_2	321.112	0.86	C20H16O4	0.2	Corylin	[321.11, 321.07, 322.11, 322.07, 303.06]
M121T355	121.0644	5.92	C8H11NO	1.9	(R)-(-)-2-Phenylglycinol	[103.05, 93.07, 121.06, 91.05, 121.1]
M237T412	237.1824	6.87	C15H24O2	0	8-Hydroxyageraphorone	[219.17, 111.08, 204.15, 237.19, 189.13]
M121T533	121.1001	8.89	C15H26O	0.9	Patchouli alcohol	[121.1, 105.07, 93.07, 91.05, 121.07]
M439T1103	439.1375	18.39	C24H22O8	1.8	Kotanin	[439.14, 277.09, 440.15, 109.78, 278.09]
M193T267	193.084	4.44	C11H12O3	1.2	Acetic acid, p-toluoyl-, methyl ester	[91.05, 193.09, 105.07, 161.06, 133.06]
M177T117	177.1005	1.95	C10H12N2O	0.6	Serotonin	[160.08, 95.01, 132.08, 141.02, 115.05]
M135T323	135.0789	5.39	C15H20O2	0.4	6-(4-Hydroxy-3-methylphenyl)-2-methylhept-2-en-4-one	[135.08, 91.05, 107.09, 115.05, 117.07]
M209T749_1	209.115	12.49	C10H18O3	1	Cyclobutyrol	[191.1, 107.05, 209.11, 135.04, 109.03]
M169T689	169.1205	11.48	C10H16O2	1.2	Geranic acid	[123.12, 109.1, 169.12, 95.08, 93.07]
M161T194	161.0579	3.23	C10H10O3	0.4	Mellein	[105.07, 133.06, 115.05, 161.06, 103.05]
M478T818_3	478.2877	13.63	C20H36N4O8	0.9	Desferrioxamine H	[337.27, 95.09, 460.28, 478.29, 306.28]
M177T268	177.0894	4.47	C16H20O5	0.2	2-Homoveratryl-4-methoxy-2,3-dihydropyran-6-one	[177.09, 146.07, 121.06, 95.01, 149.02]
M209T47_1	208.9691	0.78	C6H4Cl2N2O2	4.2	Botran	[208.97, 124.92, 191.94, 122.92, 190.96]
M171T102	171.0272	1.7	C7H6O5	2.5	Gallic acid	[127.04, 153.02, 109.03, 107.01, 125.02]
M203T601_2	203.1771	10.02	C15H26O2	0.2	(1S,4R)-7-Isopropyl-1,4-dimethyl-2,3,3a,5,6,8a-hexahydroazulene-1,4-diol	[203.18, 133.1, 147.12, 105.07, 119.09]
M107T479	107.0847	7.99	C10H16O	3.2	.alpha.,.beta.-Thujone	[91.05, 107.09, 105.07, 109.07, 108.08]
M231T221	231.1103	3.68	C13H14N2O2	0.4	Tetrahydroharman-3-carboxylic acid	[158.1, 188.07, 214.09, 231.11, 130.07]
M235T40	234.9586	0.67	C8H6BrFO2	0.9	2-Bromo-6-fluoro-3-methylbenzoic acid	[216.95, 234.96, 188.96, 160.96, 198.94]
M175T407	175.1467	6.78	C13H20O	0.5	4-(2,4,6-Trimethyl-3-cyclohexen-1-yl)-3-buten-2-one	[175.15, 119.09, 133.1, 105.07, 175.11]
M187T561	187.1466	9.35	C14H26O4	0.5	Tetradecanedioic acid	[187.15, 145.1, 119.09, 131.09, 159.12]
M133T50_2	133.0958	0.84	C5H12N2O2	0.4	2-Amino-3-(dimethylamino)propionic acid	[116.07, 115.09, 133.1, 134.06, 110.54]
M235T545	235.1667	9.08	C15H24O3	0.4	Ageratriol	[235.17, 121.1, 119.09, 217.16, 175.15]
M443T320	443.1474	5.34	C22H28O7	1.1	Asteltoxin C	[443.15, 411.13, 425.14, 444.15, 393.12]
M204T244	204.1223	4.07	C10H13N5	0.3	Isopentenyladenine	[136.06, 204.12, 148.06, 204.09, 126.06]
M257T49	257.0716	0.82	C9H12N4O3S	4.9	Acetanilide, 4′-(amidinosulfamoyl)-	[257.07, 140.07, 257.14, 140.9, 198.86]
M344T199	344.1301	3.32	C15H18O8	1	4-O-D-Glucopyranosyl-p-coumaric acid	[147.04, 165.05, 119.05, 344, 194.97]
M247T174_2	247.1301	2.91	C15H22O5	0.2	Dihydrophaseic acid	[247.13, 105.07, 229.12, 95.05, 119.09]
M141T133	141.0532	2.22	C7H8O3	4.2	Gentisyl alcohol	[141.05, 126.03, 113.96, 141.02, 95.01]
M211T362_2	211.1682	6.03	C19H32O7	0	9.xi.-O-.beta.-D-Glucopyranosyloxy-5-megastigmen-4-one	[137.1, 109.1, 175.15, 211.17, 135.12]
M203T375	203.1773	6.25	C15H26O2	0.7	1,5-Naphthalenedimethanol, 1,2,3,4,4a,5,8,8a-octahydro-1,4a,6-trimethyl-	[203.18, 95.09, 147.12, 203.05, 105.07]
M123T324	123.0799	5.39	C12H18O	1.6	1,4a-Dimethyl-4,4a,5,6,7,8-hexahydro-2(3H)-naphthalenone	[123.08, 123.06, 95.09, 95.05, 123.04]
M171T212	171.0982	3.53	C20H24N2O3	3.6	3-Hydroxyquinidine	[171.1, 144.08, 107.08, 93.07, 154.06]
M135T60	135.0015	0.99	C6H2N2S	2.9	3,4-Dicyanothiophene	[135, 136.02, 107.08, 136.06, 135.06]
M146T61	146.0908	1.02	C5H11N3O2	0.3	4-Guanidinobutyric acid	[146.09, 130.05, 104.07, 146.12, 128.08]
M144T513	144.1368	8.56	C8H17NO	0.7	4-sec-Butylmorpholine	[144.14, 145.1, 102.09, 144.08, 99.04]
M235T408	235.1667	6.79	C24H32O5	0.7	10-Hydroxy-1,6-dimethyl-9-(propan-2-yl)-5,12-dioxatricyclo [9.1.0.04,6]dodecan-8-yl (2E)-3-phenylprop-2-enoate	[235.17, 133.1, 217.16, 105.07, 119.09]
M421T901	421.3476	15.02	C47H74O17	2.7	(3.beta.,5.Xi.,9.Xi.,18.Xi.,22.beta.)-22-Hydroxy-25-oxoolean-12-en-3-yl 6-deoxy-.alpha.-L-mannopyranosyl-(1->2)-.beta.-D-xylopyranosyl-(1->2)-.beta.-D-glucopyranosiduronic acid	[421.32, 117.09, 422.33, 113.13, 133.08]
M213T328	213.1251	5.47	C15H20O3	0.1	Parthenolide	[213.13, 195.12, 180.09, 165.07, 170.11]
M141T108	141.0521	1.8	C7H8O3	0.3	3-Methoxycatechol	[109.03, 113.96, 95.01, 141.02, 141.05]
M419T194	419.1475	3.24	C23H24O6	2.8	Dimethoxycurcumin	[419.15, 317.08, 420.15, 229.07, 318.08]
M249T265	249.1094	4.42	C14H16O4	0.2	7,8-Dihydrokawain-5-ol	[249.11, 159.08, 187.08, 129.07, 131.09]
M144T117	144.064	1.94	C6H13NO5	1	D-Glucosamine	[144.05, 99.04, 113.03, 126.04, 127.04]
M146T163	146.1161	2.72	C7H15NO2	0.7	N-Methylisoleucine	[100.11, 146.06, 100.05, 146.08, 128.05]
M601T173	601.1252	2.88	C27H30O13	0.5	MCULE-3949449025	[601.13, 311.05, 449.08, 602.13, 188.07]
M461T433_2	461.1941	7.22	C26H30O6	1.2	Isosophoranone	[461.19, 317.08, 462.2, 229.07, 185.04]
M217T371	217.1564	6.18	C15H24O3	0.4	6-Hydroxy-4-(hydroxymethyl)-3,4a,8,8-tetramethyl-5,6,7,8a-tetrahydro-4H-naphthalen-1-one	[217.16, 202.14, 145.1, 111.08, 135.08]
M144T438	144.1368	7.29	C8H17NO	0	2-(1-Piperidyl)propan-2-ol	[144.14, 99.04, 144.08, 145.1, 102.09]
M483T174	483.1471	2.9	C20H28O12	0.6	6-O-(Phenylacetyl)-.alpha.-D-glucopyranosyl .alpha.-D-glucopyranoside	[483.15, 321.1, 339.11, 363.11, 303.09]
M175T51	175.1173	0.85	C12H25N5O3	0.5	D-Leucyl-L-arginine	[175.12, 116.07, 130.1, 158.09, 112.09]
M126T1013	126.101	16.88	C6H11N3	1.2	3-Methylhistamine	[126.1, 127.09, 109.08, 126.07, 127.04]
M219T175	219.1356	2.91	C15H20O4	0.6	Antheindurolide A	[219.14, 173.13, 159.12, 119.09, 131.09]
M735T527	735.3547	8.78	C36H56O14	2.2	Acobioside A	[735.35, 563.32, 545.31, 527.3, 689.35]
M191T279	191.1046	4.65	C12H14O2	0.1	Ligustilide A	[191.11, 173.1, 145.1, 191.07, 133.1]
M301T289_3	300.9952	4.82	C14H6O8	4.9	Ellagic acid	[301, 302, 229.01, 283.99, 299.99]
M180T137	180.013	2.28	C8H9NO3S	3	N-Acetylbenzenesulfonamide	[180.03, 116.07, 132.05, 181.03, 117.07]
M265T906	265.1446	15.11	C15H22O4	1.4	Aspterric acid	[96.96, 265.14, 265.21, 266.15, 96.97]
M166T52	166.015	0.87	C7H5NO4	3.3	Quinolinic acid	[166.02, 106.98, 124, 102.05, 136]
M337T187	337.1462	3.12	C21H22O4	4.5	Licochalcone A	[337.15, 113.02, 338.15, 101.06, 112.93]
M335T209	335.1305	3.48	C21H20O4	5	8-Cinnamoyl-5,7-dihydroxy-2,2,6-trimethylchromene	[335.13, 113.02, 160.04, 159.1, 336.13]
M311T728	311.1649	12.13	C20H24O3	1	Hypolide	[311.17, 183.01, 184.02, 312.17, 216.01]
M223T152	223.0217	2.54	C12H12FN3OS	1.8	4-Allyl-3-[(4-fluorophenoxy)methyl]-1H-1,2,4-triazole-5-thione	[223.02, 221.01, 193.01, 129.02, 224.03]
M337T55	337.0733	0.91	C19H14O6	3.3	Rabelomycin	[337.07, 115, 175.02, 174.01, 338.08]
