# Supplementary material for: Mechanistic Insights into Active Components of Rosa Roxburghii Juice Against Fluoride-Induced Osteoarthritis
Source: Antioxidants (Basel). 2026 Feb 28;15(3):309. doi: 10.3390/antiox15030309 (PMC13023644; doi:10.3390/antiox15030309)
Supplement: Supplementary file 1 [file antioxidants-15-00309-s001.zip › Supplementary_Figure_Captions.pdf]

Supporting Information for

Mechanistic Insights into Active Components of Rosa Roxburghii Juice Against Fluoride-Induced Osteoarthritis

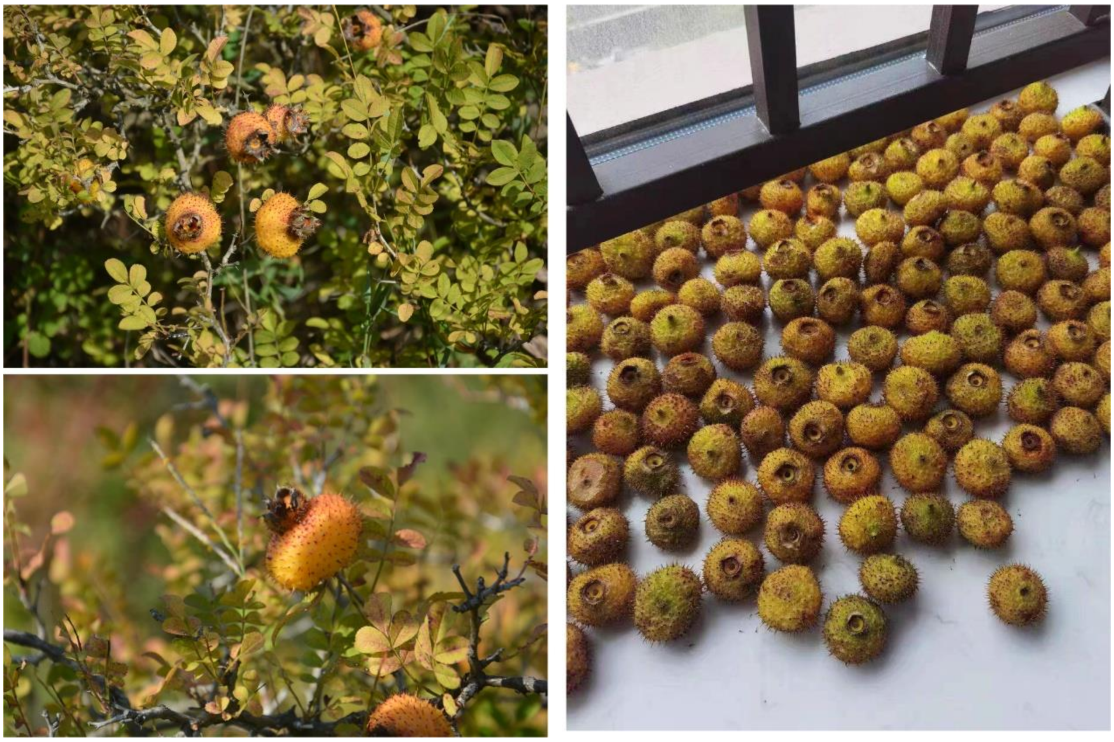

Supplementary Figure S1. Representative photograph of fresh fruits of *Rosa roxburghii* Tratt. cultivar 'Guinong 5' used in this study.

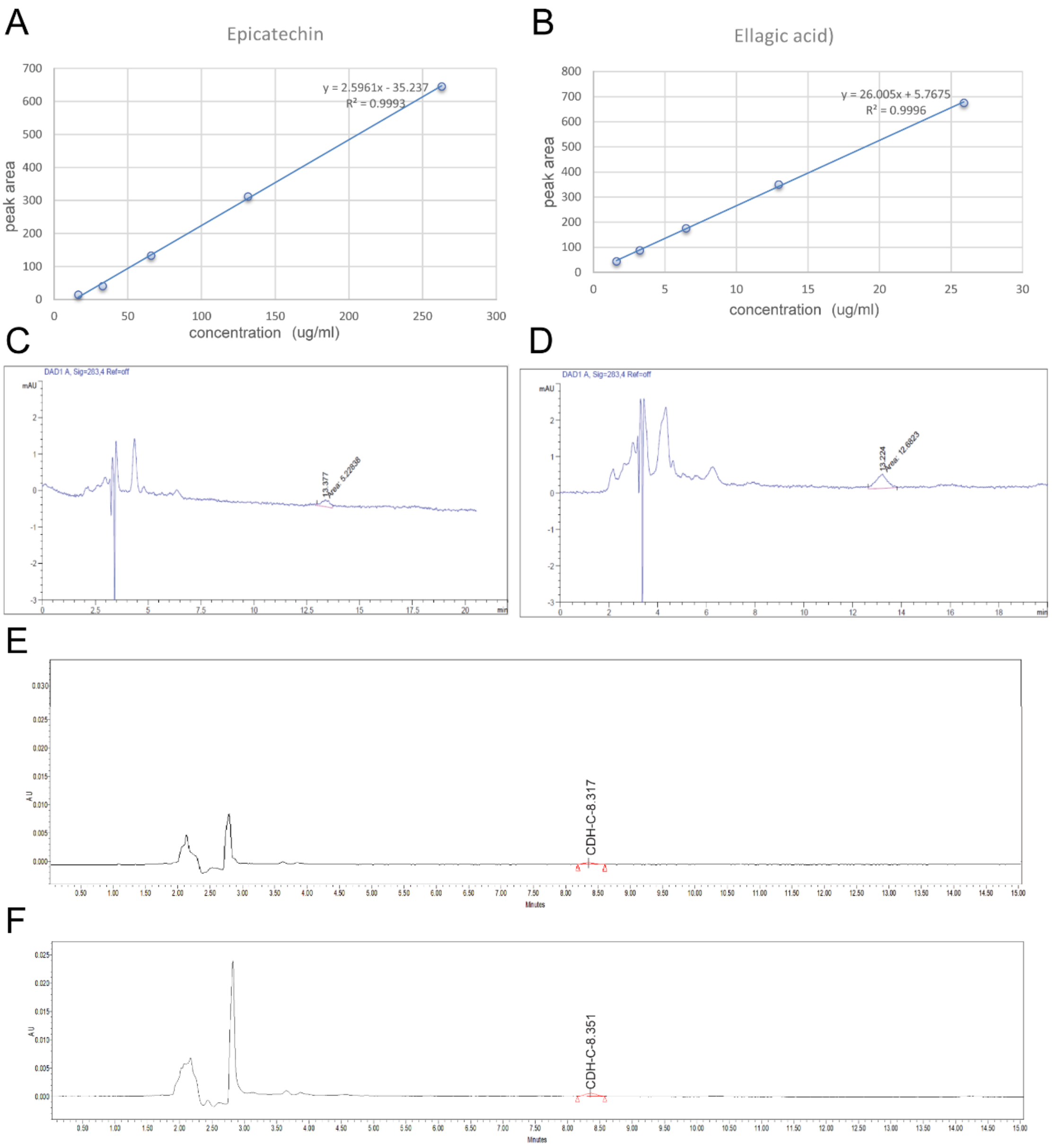

Supplementary Figure S2. Validation of the HPLC-DAD method for epicatechin and ellagic acid.(A) Calibration curve of epicatechin showing the linear relationship between peak area and concentration.(B) Calibration curve of ellagic acid showing the linear relationship between peak area and concentration.(C) Determination of the limit of detection (LOD) for epicatechin based on a signal-to-noise (S/N) ratio of 3.(D) Determination of the limit of quantification (LOQ) for epicatechin based on a signal-to-noise (S/N) ratio of 10.(E) Determination of the limit of detection (LOD) for ellagic acid based on a signal-to-noise (S/N) ratio of 3.(F) Determination of the limit of quantification (LOQ) for ellagic acid based on a signal-to-noise (S/N) ratio of 10.

All experiments were performed in triplicate. Calibration curves were constructed using six concentration levels, and linear regression analysis was applied to calculate correlation coefficients (R^2).
