# Supplementary figures and images for: Mechanistic Insights into Active Components of Rosa Roxburghii Juice Against Fluoride-Induced Osteoarthritis
Source: Antioxidants (Basel). 2026 Feb 28;15(3):309. doi: 10.3390/antiox15030309 (PMC13023644; doi:10.3390/antiox15030309)

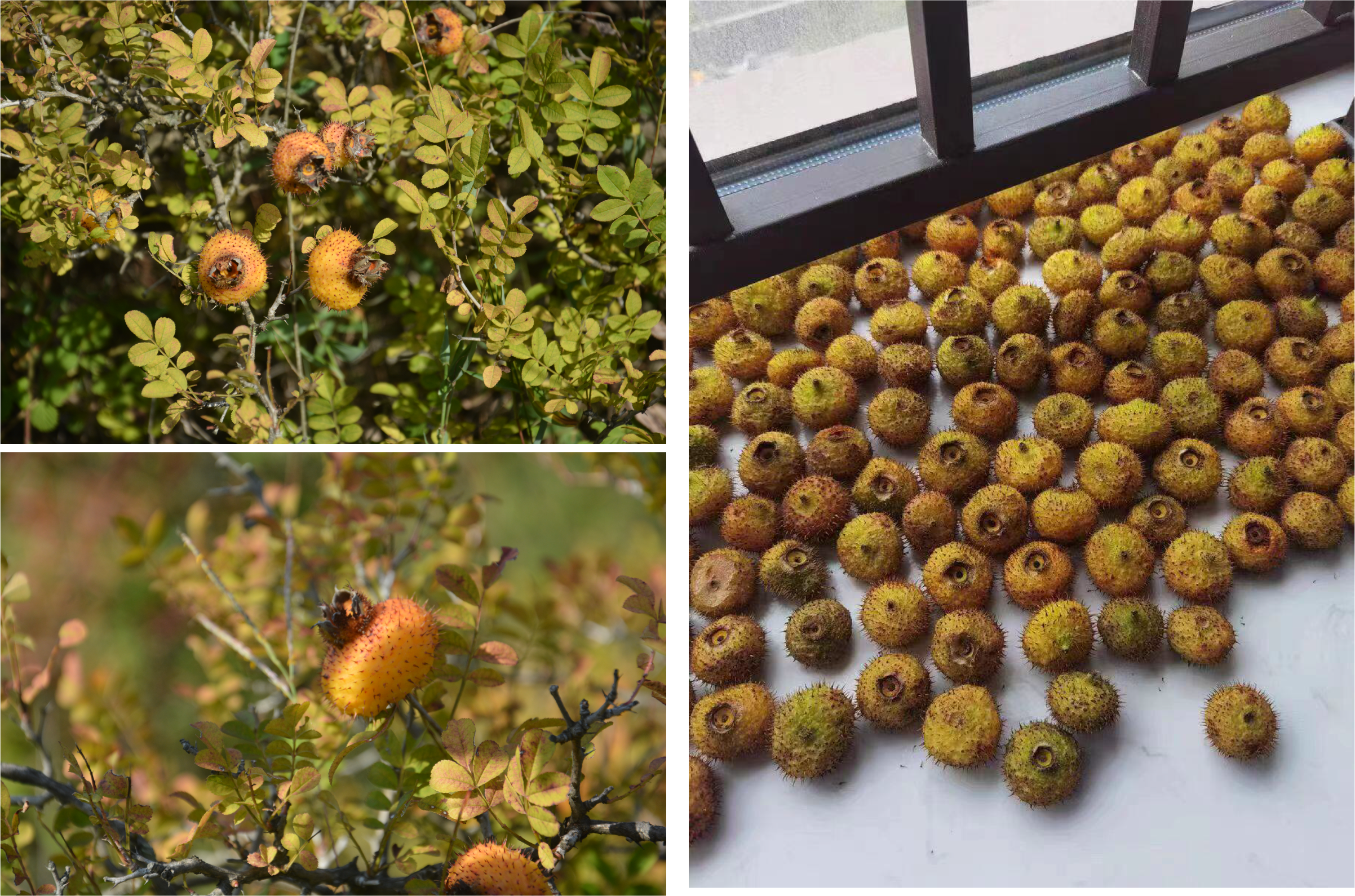

Supplement: Supplementary file 1 [file antioxidants-15-00309-s001.zip › Supplementary Figure S1.tif]

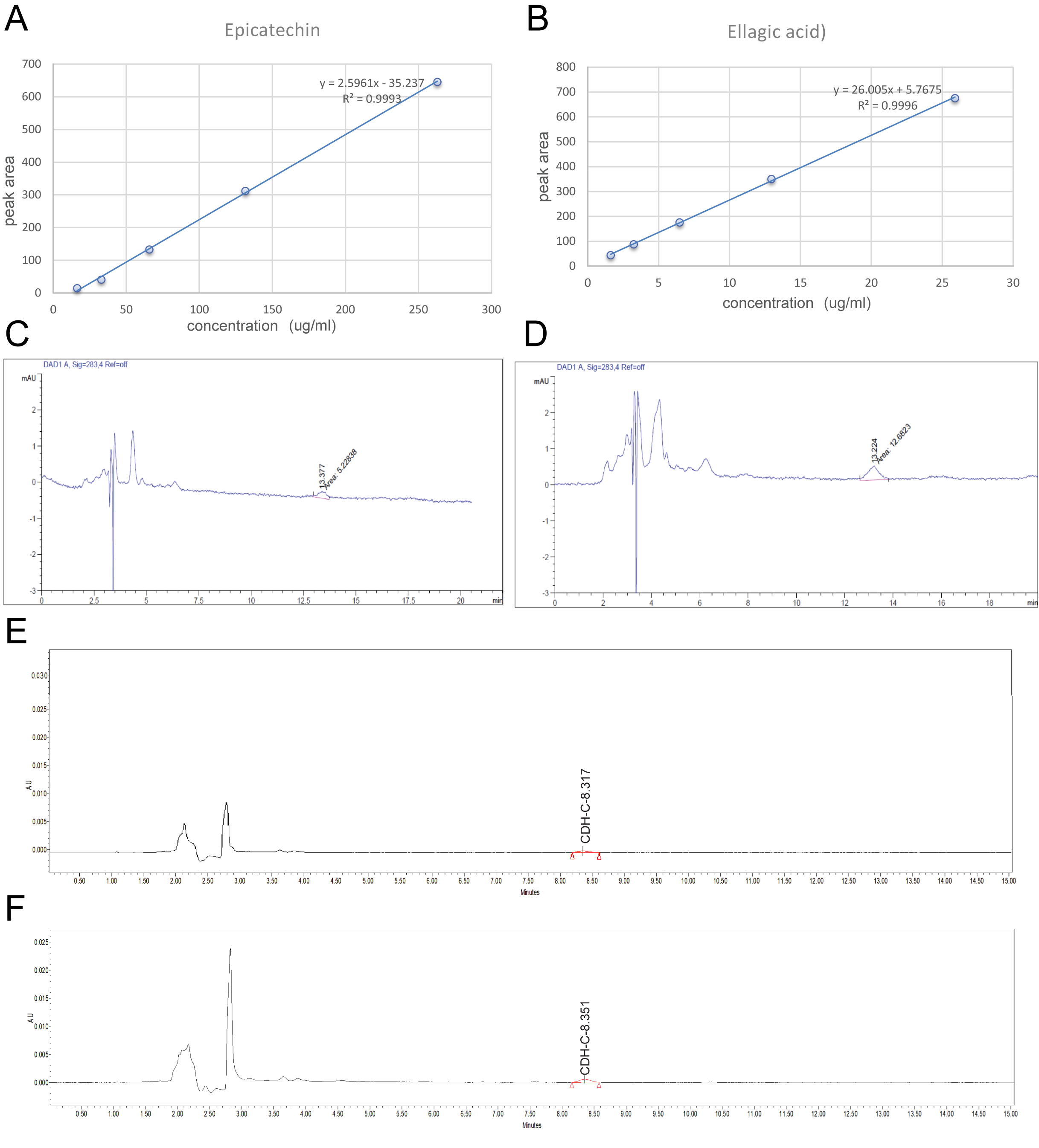

Supplement: Supplementary file 1 [file antioxidants-15-00309-s001.zip › Supplementary Figure S2.tif]
